# The ELIXIR Biodiversity Community: Understanding short- and long-term changes in biodiversity

**DOI:** 10.12688/f1000research.133724.2

**Published:** 2024-05-22

**Authors:** Robert M. Waterhouse, Anne-Françoise Adam-Blondon, Bachir Balech, Endre Barta, Physilia Ying Shi Chua, Valeria Di Cola, Katharina F. Heil, Graham M. Hughes, Lars S. Jermiin, Matúš Kalaš, Jerry Lanfear, Evangelos Pafilis, Patricia M. Palagi, Aristotelis C. Papageorgiou, Joana Paupério, Fotis Psomopoulos, Niels Raes, Josephine Burgin, Toni Gabaldón

**Affiliations:** 1 Department of Ecology and Evolution, SIB Swiss Institute of Bioinformatics, Universite de Lausanne, Lausanne, Vaud, 1015, Switzerland; 2 INRAE, BioinfOmics, Plant Bioinformatics Facility, Universite Paris-Saclay, Gif-sur-Yvette, Île-de-France, 78026, France; 3 Istituto di Biomembrane, Bioenergetica e Biotecnologie Molecolari, Bari, 70126, Italy; 4 Institute of Genetics and Biotechnology, Magyar Agrar- es Elettudomanyi Egyetem, Gödöllő, Pest County, Hungary; 5 ELIXIR, Wellcome Genome Campus, Hinxton, England, CB10 1SD, UK; 6 SIB Swiss Institute of Bioinformatics, Lausanne, Vaud, 1015, Switzerland; 7 School of Biology and Environmental Science, University College Dublin, Dublin, Leinster, Ireland; 8 Systems Biology Ireland, School of Medicine, University College Dublin, Dublin, Leinster, Ireland; 9 School of Mathematical and Statistical Sciences, University of Galway, Galway, Ireland; 10 Department of Informatics, Universitetet i Bergen, Bergen, Hordaland, Norway; 11 Institute of Marine Biology, Biotechnology and Aquaculture, Hellenic Centre for Marine Research, Heraklion, 71003, Greece; 12 Department of Molecular Biology and Genetics, Democritus University of Thrace, Alexandroupolis, Greece; 13 EMBL-EBI, Wellcome Genome Campus, Hinxton, England, CB10 1SD, UK; 14 Institute of Applied Biosciences, Centre for Research and Technology Hellas, Thessaloniki, Greece; 15 Naturalis Biodiversity Center, Leiden, South Holland, The Netherlands; 16 Institut de Recerca Biomedica, Barcelona, Catalonia, Spain; 17 Centro Nacional de Supercomputacion, Barcelona, Catalonia, Spain

**Keywords:** White Paper, ELIXIR Strategy, Community Roadmap, Biodiversity Networks, Biodiversity Informatics, Environmental Systems, Data Science

## Abstract

Biodiversity loss is now recognised as one of the major challenges for humankind to address over the next few decades. Unless major actions are taken, the sixth mass extinction will lead to catastrophic effects on the Earth’s biosphere and human health and well-being. ELIXIR can help address the technical challenges of biodiversity science, through leveraging its suite of services and expertise to enable data management and analysis activities that enhance our understanding of life on Earth and facilitate biodiversity preservation and restoration. This white paper, prepared by the ELIXIR Biodiversity Community, summarises the current status and responses, and presents a set of plans, both technical and community-oriented, that should both enhance how ELIXIR Services are applied in the biodiversity field and how ELIXIR builds connections across the many other infrastructures active in this area. We discuss the areas of highest priority, how they can be implemented in cooperation with the ELIXIR Platforms, and their connections to existing ELIXIR Communities and international consortia. The article provides a preliminary blueprint for a Biodiversity Community in ELIXIR and is an appeal to identify and involve new stakeholders.

## Introduction

### Biodiversity threats and challenges

Biological diversity—or biodiversity—refers to the variety and variability of life on Earth, encompassing genetic and species diversity at the levels of populations, communities, and ecosystems. Biodiversity reflects the ever-changing natural balance that has evolved over billions of years, sustaining communities of interdependent and interacting organisms. Those balances form the basis of a healthy Earth, including the ecosystem functions that support human well-being ( *i.e.*, ecosystem services). With growing demands on nature due to human activities, the Anthropocene is upsetting this balance and is consequently witnessing an unprecedented loss of biodiversity globally ( WWF, 2022; Johnson *et al.*, 2017). These declines pose a grave threat to humanity, the severity of which is increasingly recognised by international organisations, regional bodies, national governments, and society. The urgency to act is recognised particularly in the field of conservation biology, which has been described as a “discipline with a deadline” ( Soulé & Wilcox, 1980).

Strategies to protect and restore biodiversity are wide-ranging in scope and scale, with policies and actions that require broad support to be feasible and effective *e.g.*, goals 12-15 of the 17 Sustainable Development Goals (SDGs) adopted by the United Nations ( UN, 2015). Biodiversity research aimed at building the knowledge and resources that inform management practices and policy is equally wide-ranging, often bringing together researchers from different disciplines, such as taxonomists, ecologists, evolutionary biologists, and informaticians. This is particularly true for the growing field of interdisciplinary research taking advantage of molecular sequence data, which recognises the relevance of and advantages offered by genetic and genomic data in biodiversity assessment, monitoring, conservation, and restoration ( Hoban *et al.*, 2021; Lewin *et al.*, 2022). Connecting such molecular sequence data with biodiversity research infrastructures (see *Extended Data* ( Waterhouse, 2023)) and resources is a critical step towards facilitating exchange of knowledge, sharing, and interoperability of large and complex datasets ( Waterhouse *et al.*, 2022).

As a European life sciences infrastructure, ELIXIR strives to coordinate bioinformatics resources from across Europe to enable researchers to access and analyse life science data, to improve the value and impact of life science research on public health, the environment, and the economy. The need for informatics solutions to address key societal challenges inspires many scientists from across the ELIXIR Nodes to increasingly engage in different aspects of biodiversity research. This stems from a natural alignment with ELIXIR’s overarching mission to support the management of public research data, integrate and coordinate life science resources, and foster the development of innovative services and technical solutions in Europe ( Harrow *et al.*, 2021). Here we present the ELIXIR Biodiversity Community, comprised of researchers from different disciplines, united by a shared recognition of the main societal and informatics challenges, as well as key scientific and organisational opportunities; how these connect with ELIXIR Platforms and other ELIXIR Communities, as well as with the wider “ecosystem” of biodiversity projects and infrastructures; and set out our roadmap for building on ELIXIR expertise to grow the ELIXIR Biodiversity Community and engage with the development of resources and infrastructures to support biodiversity research.

### Societal challenges and global responses

Biodiversity represents the variety of organisms on the planet at all taxonomic levels, a result of a long and complex evolutionary process. Biodiversity is essential for life itself, for the adaptation of populations, species, communities, and ecosystems towards rapid change in biotic and abiotic parameters, including climate change. From a human standpoint, biodiversity forms the foundation of ecosystem services that are indispensable for human well-being and a healthy planet, and has long been a source of adaptive solutions or innovations in several critical areas such as food production. Despite its importance, biodiversity has been declining at a mass-extinction-level rate ( IPBES, 2019) over the last decades. The unsustainable human development model has increased pressures on biodiversity, through climate change ( IPCC, 2022; Wezel *et al.*, 2020), invasive species, habitat loss and degradation, and the depletion of natural resources ( IPBES, 2019). The decline of biodiversity at this rate often creates unpredictable threats and changes to ecological oscillations, such as the increased risk of new human diseases ( Frumkin & Haines, 2019), the collapse of ecosystem services, the degradation of natural resources, and the increased possibility of a global food crisis ( FAO, 2019).

At the same time, scientists and naturalists do not even know what is being lost, as around 80% of biodiversity at the species and population levels remains undescribed and/or underrepresented in inventories and databases ( Mora *et al.*, 2011; Costello *et al.*, 2013; Moura & Jetz, 2021; Bispo *et al.*, 2022; Boekhout *et al.*, 2022; Chimeno *et al.*, 2022). Furthermore, most research and monitoring efforts tend to focus on a limited number of biodiversity levels or elements. While there is significant literature around biodiversity loss ( *e.g.,* a Scopus query [13.09.2022] for “biodiversity loss” returns 33,324 documents), there is a very limited effort in reviewing biodiversity using high-throughput data (Scopus query [13.09.2022] for “Biodiversity loss” AND (“omics” OR “genomics” OR “metagenomics”) returns only 1,795 documents). This clearly indicates a bias in reporting, which has repercussions on the decision-making process pertaining to biodiversity conservation efforts ( Gadelha *et al.*, 2021). This brings forward an additional challenge of shifting perspectives from narrow, low-throughput efforts towards more holistic, high-throughput initiatives, including better citizen scientist contributions towards these efforts. Humanity may miss important solutions to key problems for its survival, such as the loss of important genetic variants among wild plants, animals, and microbes for agriculture ( Nic Lughadha *et al.*, 2020) and for dealing with health issues ( Marselle *et al.*, 2021).

Following the 1992 United Nations Convention on Biological Diversity (CBD), governments and international organisations have responded to the decline of biodiversity with policies, and restoration and protection strategies. However, the initial goals of these have not been reached and biodiversity decline continues accelerating ( IPBES, 2019; Turvey & Crees, 2019; WWF, 2022). For the new targets set by the post-2020 global biodiversity framework ( GBF, 2023) to succeed, research is considered to be key, especially the interaction between science, society, and policy makers ( Blicharska *et al.*, 2019; Hermoso *et al.*, 2022; Nature, 2022), with net improvements by 2050 to achieve the CBD’s vision of “living in harmony with nature by 2050”. Today, scientists recognise the important roles that genetic and genomic data can play in biodiversity discovery, assessment, monitoring, conservation, and restoration, to ensure the long-term resilience of ecosystems ( Hoban *et al.*, 2020; Gadelha *et al.*, 2021; Segelbacher *et al.*, 2022; Formenti *et al.*, 2022; Theissinger *et al.*, 2023). The contribution of genomics and bioinformatics towards these targets, and many of the associated technical and scientific challenges are described in Waterhouse *et al.* (2022), together with the possible contribution of the ELIXIR European Strategy Forum for Research Infrastructures to meet them.

### Scientific opportunities in biodiversity research

Biodiversity researchers are increasingly realising the potential offered by modern technologies, particularly in genomics, to create new opportunities for developing tools and resources that will transform the field. These opportunities lie primarily in the types of scientific applications that are becoming more feasible and scalable through continued advances in genomics technologies alongside enhanced data management systems. A long-term vision sees a future where sequence-based biodiversity monitoring at scale becomes a default and provides the means for ecosystem biodiversity characterisation in space and time, complemented and enhanced by other biomonitoring technologies. In support of realising these opportunities, ongoing global and regional efforts are building capacity to generate catalogues of reference DNA barcodes (International Barcode of Life, iBOL) ( Hobern, 2021) and genomes by the Earth BioGenome Project (EBP) ( Lewin *et al.*, 2018, 2022) as well as the European Reference Genome Atlas ( ERGA, 2023), or both by the Biodiversity Genomics Europe ( BGE, 2023; Mazzoni *et al.*, 2023) project. Along with this increased production, concurrent development of the necessary tools and resources will greatly enhance our abilities to:

• Maintain and query increasingly comprehensive reference DNA barcode and genome catalogues, improving taxonomic coverage and differentiation (including of cryptic species), and coordinating the efforts of various initiatives under global and regional umbrellas *e.g.*, McGee *et al.* (2019);
• Connect and integrate these molecular resources with other biodiversity data (traits, observations, literature, *etc.*) *e.g.*, König *et al.* (2019), using an increasingly standardised and harmonised taxonomic framework as the common backbone;
• Use these integrated resources for applied data-driven science to understand the diversity of extant life on Earth, how that diversity functions and interacts, and how it responds to changing environmental pressures ( Pereira *et al.*, 2012);
• Implement monitoring of lesser-known or complex ecosystems, including for enhancing understanding of species interactions and dynamics, as well as for species discovery and exploration of “dark taxa” *e.g.,* Rahman *et al.* (2022);
• Include assessments of within-species, population-level genetic diversity to support characterisations of their evolutionary histories and predictions of their future prospects in the face of ongoing climatic changes ( Pearman *et al.*, 2024);
• Operationalise the assessment of Essential Biodiversity Variables (EBVs) across taxa and spatiotemporal scales, focusing on species distribution and abundance ( Kissling *et al.*, 2018; Jetz *et al.*, 2019);
• Engage with naturalists and citizen scientist groups through the use of new technologies that help build a democratised monitoring framework and improve characterisation of ecosystem biodiversity in space and time ( Robinson & Peres, 2021);
• Evaluate biodiversity declines, as well as population-level adaptation and migration processes, in the context of anthropogenic activities ( *e.g.*, climate change and urbanisation consequences, ( Finn *et al.*, 2023)), and understand key aspects necessary to restore ecosystem functions ( Breed *et al.*, 2019) to help prioritise biodiversity conservation, restoration, and “rewilding” efforts ( *e.g.,* particularly relevant to at-risk biodiversity hotspots).

### Organisational opportunities and ELIXIR’s roles

The field of biodiversity assessment and research, from an organisational context, is broad, complex, and distributed. There are a multitude of organisations that operate across international borders, within countries, and at a local level (see *Extended Data* ( Waterhouse, 2023)). This landscape is further demarcated along scientific and technical lines, with organisations that focus on taxonomies, ecology, molecular sciences, and method development (necessitated by the increasingly large and complex amount of data being generated). ELIXIR, perhaps uniquely, stands as a hub for the molecular sciences and bioinformatics at an international and national level across many scientific disciplines ( Waterhouse *et al.*, 2022). Biodiversity research and infrastructures increasingly rely on molecular data ( Karp *et al.*, 1997; Porter & Hajibabaei, 2018), so ELIXIR is well placed to lead organisational alignments and collaborations: from a core set of partners across Europe mainly within the field of molecular sciences, to an expanding variety of partner organisations that focus on other biodiversity-related research and resources (see below for examples from the ecosystem of biodiversity projects, resources, and infrastructures). Importantly, this extends beyond the data themselves as FAIRification of digital research objects ( Wilkinson *et al.*, 2016), championed by ELIXIR’s Services and Platforms, is increasingly recognised as essential in biodiversity research ( Wetzel *et al.*, 2018; Lannom *et al.*, 2020). Opportunities to help coordinate and align organisational activities in the biodiversity domain arise naturally from ELIXIR’s established European-wide “network of networks” approach, connecting to existing initiatives at both the national and international levels. With ELIXIR’s strengths in molecular sciences, a “hub and spokes” model would help augment opportunities to connect molecular-focused bioinformatics tools, protocols, and resources with the many other biodiversity-related infrastructure and stakeholder organisations. Building on these strengths in data science and a connected network across Europe, ELIXIR can contribute to coordinated efforts designed to support and grow the many existing initiatives in the domains of biomonitoring, ecosystem health, and biodiversity research.

### Informatics challenges facing biodiversity infrastructures and resources

The variety of existing biodiversity data infrastructures and resources is a testament to the long-standing recognition by multiple stakeholders of their importance, currently reflected in the growing European and global commitments to prevent further biodiversity decline and ensure the long-term health of ecosystem services. This heterogeneity, however, gives rise to many challenges, both technical in terms of data analysis (due to inadequacies of existing methodologies), data integration and data interaction, and at the level of the scientific community, which faces a heterogeneous landscape of infrastructures and resources that can be difficult to navigate ( Blaxter & Floyd, 2003; Huang *et al.*, 2012; Levin *et al.*, 2014). The methodological and logistical challenges range from scaling up (needed to be able to process the increasing amounts of complex molecular data) to the management of these data and working on connecting them to other biodiversity research infrastructures ( Waterhouse *et al.*, 2022). The biodiversity research community needs to proactively seek common solutions that enable molecular technologies to advance biodiversity research. A key part of this is the building of distributed infrastructures for life-science data that avoid or minimise unnecessary duplication of effort to be able to advance efficiently towards common goals. To this end, informatics solutions will need to be developed to address the practicalities of common challenges, such as:

• The need to constantly incorporate knowledge-based updates and resolve conflicts to maintain standardised taxonomies that serve as a dynamic framework that facilitates interoperability across research infrastructures;
• Building data and metadata brokering services that support coordinated community engagement to ensure good data management through technical infrastructures for aiding and automating data submission;
• Developing the means, through text mining and curation, to identify and liberate in digital form invaluable historical or baseline data trapped in the literature (including those published in non-English sources), or in museum and other natural history collections;
• Improving the accessibility of research results through publications ( *e.g.,* by making published traits, tables, treatments, specimens, figures *etc.*), citable and reusable ( *e.g.,* through nanopublications), and including identifiers of cited elements (genes, specimens, taxonomic names, treatments);
• Improving and harmonising currently highly heterogeneous metadata collection standards to promote the adoption of community best practices that will maximise findability, accessibility, interoperability, and reusability of digital research objects ( *i.e.*, drive biodiversity research towards FAIR compliance);
• Scaling up of services for data and metadata management to keep pace with and accommodate the increases in data production ( *e.g.,* genomics) and collection ( *e.g.*, Essential Biodiversity Variables);
• Developing frameworks that deliver an increasingly integrated and interconnected landscape of biodiversity research infrastructures, utilising developments in application programming interfaces and Semantic Web services;
• Ensuring widespread access to high-performance computing (HPC) and HPC-deployable software and data-management systems, including containers and workflows, to enable decentralised efforts while promoting standardisation.

## The ELIXIR Biodiversity Community: An “ecosystem” of projects

ELIXIR Communities are groups of experts across ELIXIR Nodes and beyond that represent a scientific or technological theme which drives the development of standards, services, and/or training in and across services offered by ELIXIR, thereby connecting the infrastructure services to research domains ( Heil & Garrard, 2024). The ELIXIR Biodiversity Community was first launched in 2019 as a Focus Group to develop and coordinate ELIXIR Nodes’ tools, resources, and research work connected to the biodiversity domain. As part of the process of maturing from a Focus Group to a Community, members initiated activities including: (1) cataloguing ELIXIR Services that support biodiversity research; (2) developing and publishing their “Recommendations for connecting molecular sequence and biodiversity research infrastructures through ELIXIR” ( Waterhouse *et al.*, 2022); (3) coordinating ELIXIR Node participation in Horizon Europe project proposals - The Biodiversity Community Integrated Knowledge Library (BiCIKL) and Biodiversity Genomics Europe (BGE); and (4) beginning to establish connections with key external partners/projects in the biodiversity domain (such as those listed in Table 1); leading to the formal recognition in 2022 as an ELIXIR Community ( Waterhouse *et al.*, 2023).

**Table 1.** Summaries of a selection of transnational and national biodiversity-related projects in which ELIXIR Nodes are involved.

Project	Node/Funder	Summary details/description
ARISE	Netherlands	ARISE (Authoritative and Rapid Identification System for Essential biodiversity information) is a digital infrastructure with a mission to provide semi-automated identification of all multicellular species in the Netherlands ( van Ommen Kloeke *et al.*, 2022).
BiCIKL	E.C. (coordinated by Pensoft)	BiCIKL (Biodiversity Community Integrated Knowledge Library) will catalyse a culture change in the way biodiversity data is identified, linked, integrated and re-used across the research cycle. We will cultivate a more transparent, trustworthy and efficient research ecosystem.
Biodiversity Genomics Europe ( BGE)	E.C. (coordinated by Naturalis Biodiversity)	By bringing together Europe’s key practitioners in two fundamental DNA-based technologies - DNA barcoding and genome sequencing - the BGE consortium aims to streamline the rollout of these methods across Europe.
Biodiversity Digital Twin ( BioDT)	E.C. (coordinated by CSC – IT CENTER FOR SCIENCE LTD.)	The Biodiversity Digital Twin prototype will provide advanced models for simulation and prediction capabilities, through practical use cases addressing critical issues related to global biodiversity dynamics.
Curated collections of DNA barcode marker	Italy	A reference collection of COXI mitochondrial DNA genes based on the integration of sequence and taxonomy data of BOLD and ENA ( Balech *et al.*, 2022).
e-BioDiv	Switzerland	Open Biodiversity FAIR-ification Services for Biospecimens stored in Swiss Natural History Museums
Earlham Institute Barcoding the Broads	UK	A Wellcome-funded programme of public engagement events and activities to explore biodiversity on the Norfolk Broads, led by the Earlham Institute as part of the work on the Darwin Tree of Life project.
ELIXIR Norway	Norway	Dedicated national ELIXIR Node funding (2022-2026) includes a focus on biodiversity and connections to other biodiversity infrastructures and projects in Norway ( *e.g.*, the Earth BioGenome Project Norway: EBP-Nor).
Establishment of an ELIXIR Contextual Data Clearinghouse	ELIXIR (Implementation study)	The objective is to develop and deploy an “ELIXIR Contextual Data Clearinghouse” for extending, correcting and improving publicly available annotations on records in sample and sequencing data resources.
Molecular Biodiversity Greece Community (MBGC)	Greece	Greece is a biodiversity hotspot and to this end, a network of networks covering different disciplines of molecular biodiversity research has been developed. MBGC aims to channel the flow of information amongst researchers, institutions, policy makers, stakeholders and local communities, remaining aligned to all relevant initiatives and infrastructures, at the national, European, and global level.
NFDI4Biodiversity	Germany	Network of diverse biodiversity data (not only molecular). Data are provided by research organisations and projects ( *e.g.*, GBOL), public authorities, professional societies and citizen initiatives. Data Management oriented. The production of the data itself is done through use cases.
Phylogenetic methodology	Ireland	A range of analytical tools is being developed to augment the bioinformatics tool kit for comparative genome analysis.
Pole National de Données de Biodiversité	France	National centre of data on biodiversity: the data are provided by the same diversity of channels as in Germany and the role of PNDB is to support FAIR data management.

Operationally, monthly online meetings coordinated by the Community co-leads with support from the ELIXIR Hub serve as the primary forum for interactions, complemented by discussions and notifications on the ELIXIR Slack Workspace’s Biodiversity Community channel. These include sharing information on members’ participation in ongoing or planned biodiversity-related projects and initiatives, including the Community-led Implementation Study “Biodiversity Networks for ELIXIR”. The online meetings also feature presentations on tools and services developed by ELIXIR Nodes as well as hosting invited speakers representing key external partners/projects. The Community’s Implementation Study encompasses four key areas of work to drive Community activities: (1) to survey and catalogue Research Data Management (RDM) elements relevant to the biodiversity domain, with a focus on molecular data; (2) to catalogue, review, and categorise tools, services, and analytical workflows currently in use by ELIXIR Nodes and the wider community, that process and analyse biodiversity-related data; (3) to describe the landscape of stakeholders ELIXIR is working with or needs to better engage with to establish a “network of networks” for biodiversity research and services; and (4) to leverage the strengths of ELIXIR’s training experience to help support the growth of the Biodiversity Community through network-driven sharing of training experiences and knowledge transfer and materials. Together, these actions are serving to enhance ELIXIR’s network of networks in helping to deliver connected data to advance biodiversity research.

Tackling the biodiversity crisis at a general level is not going to be resolved through a single action, but instead requires a complex set of interacting actions that are co-dependent but usually funded separately. ELIXIR can assume a key leading role in a subset of those actions, focused on data management and the molecular sciences, where even at the level of ELIXIR, there are a multitude of funded projects at a transnational, national, and local level. In terms of informatics solutions connected to such projects, the ELIXIR Biodiversity Community is guided by themes emerging from surveying approaches by which molecular technologies are helping to inform understanding of biodiversity ( Waterhouse *et al.*, 2022): biodiversity-related and informatics infrastructures need to develop close and strategic collaborations; work on taxonomy needs to be better aligned and standardised across different infrastructures and fields of study; metadata urgently needs harmonisation and common approaches to research data management must be widely adopted; current data science solutions need to be scaled up to address the rapidly accumulating amounts of molecular data; bioinformatics support for biodiversity research needs to be made widely available and properly maintained; user training on biodiversity research tools, services, and infrastructures needs to be prioritised; and community initiatives need to be collaborative, proactive, and solution-driven. These themes come together in a complex network of interacting projects that have distinct but related aims, usually focused on establishing communities and connections and/or building new technical solutions to help with data access, storage, or analysis. ELIXIR can serve a critical function here, as a fundamental aspect of its mission is to make connections and coordinate across complex activities. Table 1 lists a subset of ongoing projects across Europe and within ELIXIR member states that illustrate the breadth of activities underway.

## Connections with ELIXIR Platforms and Communities

ELIXIR as a Research Infrastructure is structured around (technological) Platforms as well as (user) Communities. Both of these interact on an ongoing basis, mutually supporting each other’s efforts. The ELIXIR Biodiversity Community is already collaborating with some of these and aims to broaden interactions to fully leverage the available potential and resources. Some examples of current and future interactions with ELIXIR Platforms (Tools, Compute, Data, Training, and Interoperability) are:

• The Tools Platform provides services for finding software tools and web portals (Bio.tools ( Ison *et al.*, 2019), including the https://biodiversity.bio.tools subdomain to be populated by the ELIXIR Biodiversity Community), software containers (BioContainers ( da Veiga Leprevost *et al.*, 2017)), and workflows (WorkflowHub ( Goble *et al.*, 2021)); for assessing tools (OpenEBench ( Capella-Gutierrez *et al.*, 2017)); and the best practices in providing research software ( Jiménez *et al.*, 2017)). EDAM ontology enables annotation and search of tools and other research objects by application domain, task, or data ( Black *et al.*, 2022); and an extended coverage of biodiversity research concepts could be achieved *via* engagement with the Biodiversity Community.
• Specifically for the Compute Platform: User accessible compute, potentially controlled user access *via* Authentication and Authorisation Infrastructure (AAI).
• Community data-management support, and integration with ELIXIR Core and Deposition Data resources. The European Nucleotide Archive (ENA) is a critical data deposition resource for biodiversity genomics data. A concrete example of metadata management workflow is that developed between biodiversity scientists, the Data Platform, and the Biodiversity Community Integrated Knowledge Library (BiCIKL) project ( Penev *et al.*, 2021, 2022): a metadata management workflow employs the PlutoF tool for biodiversity data and metadata management ( Abarenkov *et al.*, 2010), and the ELIXIR Data Platform services.
• Networks of tool/infrastructure users and developers to augment the Training Platform offerings ( *e.g.*, with specific courses covering aspects such as: genome annotation, meta-data brokering, *etc.*) and more complete learning paths, covering entire workflows ( *e.g.,* from sequencing to annotation, possibly covered *via* Galaxy).
• A growing necessity in the biodiversity field towards connected data, as championed by the Interoperability Platform, concretely touching on resources like: RO-Crate and link to specimens, RDMkit, FAIRsharing, Bioschemas and the FAIRcookbook. The ELIXIR Biodiversity Community aims to bring together researchers producing the data, in all their varied forms, with informaticians developing interoperability solutions, to help overcome the challenges of data heterogeneity in the field.

Regarding links between the ELIXIR Biodiversity Community and other ELIXIR Communities, these are already foreseen, and a number of synergies have been clearly identified. Some examples can be found in Table 2.

**Table 2.** Examples of links between the ELIXIR Biodiversity Community and other ELIXIR Communities.

Community	Shared activities
Food & Nutrition	Conceptualisation and implementation of interoperability data models able to integrate, standardise and harmonise data from different disciplines: metagenomics, metabolomics and transcriptomics.
Galaxy	Thousands of tools, including hundreds for biodiversity and microbial/microbiome analysis, are ready to be used on publicly-accessible HPC resources, together with workflows for data processing, which can be versioned, annotated, and shared for reuse. The European Galaxy server ( https://usegalaxy.eu) offers access to 2700+ tools and workflows. Galaxy-Ecology is its subdomain piloted by the French ELIXIR Node. A training material repository ( https://training.galaxyproject.org) is open for everyone to use and contribute to, providing slides, hands-on tutorials, and other material on using Galaxy to analyse data, with 260+ tutorials in 20+ topics including ecology, microbiome, and climate. Integration of PlutoF and other biodiversity tools into Galaxy could be carried out together with the Biodiversity Community in the near future.
Microbiome	Meta-genomic workflows and data archiving. Marine sample metadata annotation guidelines.
Plant Science	Taxonomy framework; coherent/consistent metadata standards for samples (see also interoperability PF (platform), MIAPPE (Minimum Information About Plant Phenotyping Experiments)). Alignment between the MIAPPE standard and exchange formats and the relevant TDWG (Biodiversity Information Standards) standards and exchange formats. Integration and linking different plant data types.

## A global network of biodiversity projects and infrastructures

ELIXIR entered the European Strategy Forum for Research Infrastructure’s (ESFRI) first roadmap in 2006 and reached its Landmark status in 2016 ( ELIXIR, 2021). As a distributed research infrastructure, ELIXIR coordinates, integrates, and sustains bioinformatics resources across European countries and helps address the Grand Challenges across life sciences, from marine research, *via* plants and agriculture, to health research, medical sciences, and biodiversity informatics. ELIXIR provides services in seven scientific domains including “Evolution and phylogeny” and “Genes and genomes” ( https://elixir-europe.org/services) that link the activities of the ELIXIR community to the wider landscape of life-science research infrastructures (RIs) and international projects. As RIs mature and FAIRness has become the standard to achieve interoperability between RIs, it is opportune to outline the global network of interrelated projects and infrastructures, in which ELIXIR operates to maximise synergy and to avoid redundancy.

**Figure 1.**
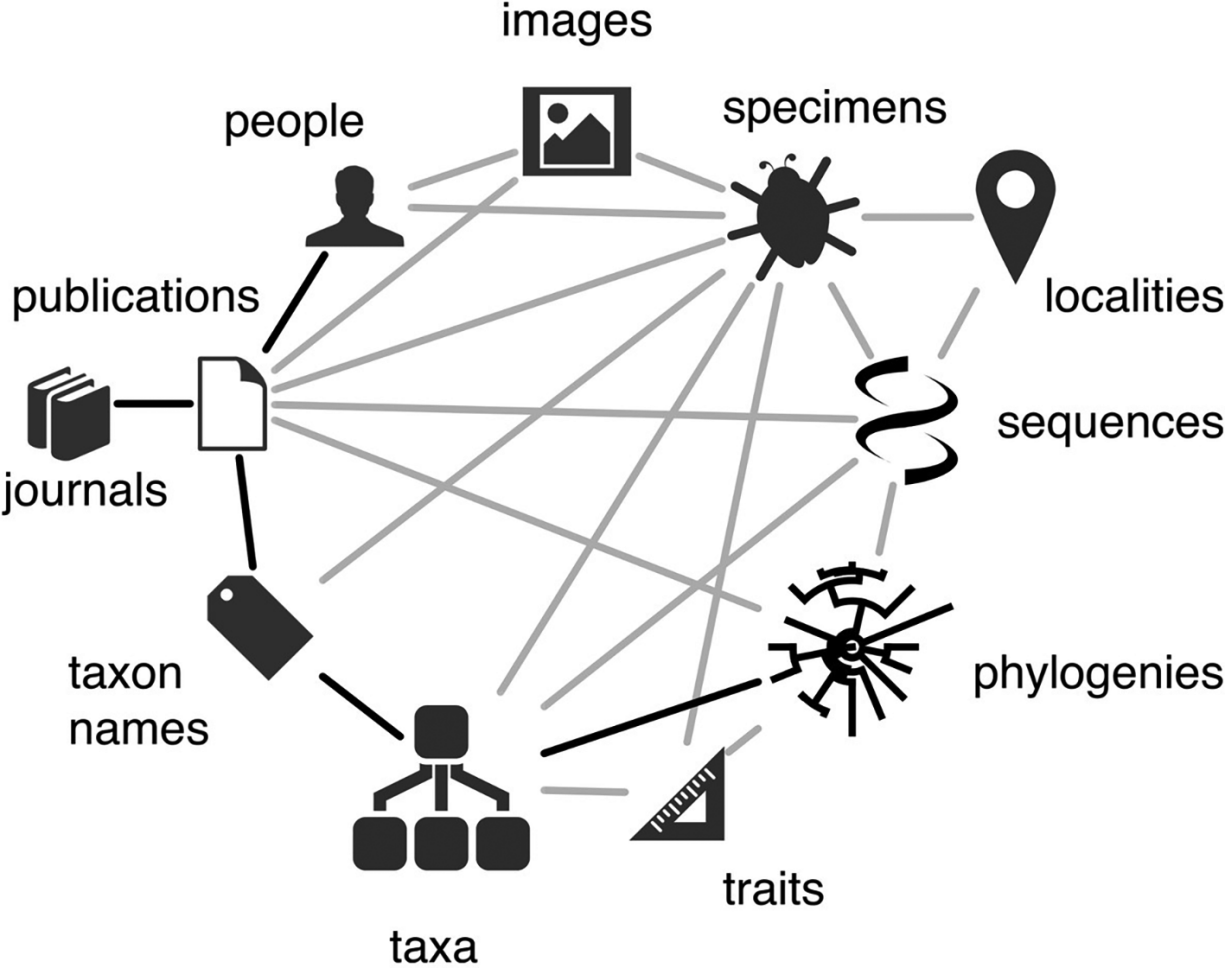
The biodiversity knowledge graph defined by Roderick D.M. Page (2013, 2016). Genomics data comprise one facet of the biodiversity knowledge graph, where questions and approaches in biodiversity research traverse the paths in this graph, and where all parts of the graph are constantly ‘evolving’ and growing. Wikimedia Commons CC-BY-4.0.

The relationships between different aspects of biodiversity data are well captured by the biodiversity knowledge graph of Roderic Page ( Figure 1). The key activities of ELIXIR are captured by the molecular domain; the biodiversity knowledge graph clearly indicates how molecular data are related to the wider spectrum of biodiversity data that are targeted by other RIs and projects. The ELIXIR Biodiversity Community benefits from connections to RIs and projects in the biodiversity domain, an overview of which can build on the landscape analyses of the ESFRI roadmaps of ESFRI 2018 ( ESFRI, 2018) and 2021 ( ESFRI, 2021), the partners of the Alliance for Biodiversity Knowledge, and the research infrastructure contact zones analysis between 10 biodiversity infrastructures, including ELIXIR ( Smith *et al.*, 2022). Additional to the data types considered by Page ( Figure 1), the contact zones analysis considers ‘observations’ and ‘collections’, or groups of specimens, as elements of the biodiversity data domain. This recognition of the variety of types of biodiversity data and the importance of integration has been key to the establishment of many RIs and research projects, for example: the Alliance for Biodiversity Knowledge; Biodiversity Genomics Europe; Biodiversity Heritage Library; Biodiversity Community Integrated Knowledge Library; iBOL BIOSCAN; Biodiversity Literature Repository; Catalogue of Life; Data Observation Network for Earth; Distributed System of Scientific Collections; Earth BioGenome Project; European Marine Biological Resource Centre; Environmental Research Infrastructures; Encyclopedia of Life; European Open Science Cloud; European Reference Genome Atlas; Europa Biodiversity Observation Network; Global Biodiversity Information Facility; Global Earth Observation System of Systems; Global Soil Biodiversity Initiative; International Barcode of Life; iNaturalist; LifeWatch ERIC; Long-Term Ecosystem Research in Europe; Microbial Resource Research Infrastructure; National Ecological Observatory Network; Open Traits Network; Plazi; Pôle national de données de biodiversité; Swiss Institute for bioinformatics Literature Services; Soil Biodiversity Observation Network; TreatmentBank; World Register of Marine Species.

In addition to the above examples of biodiversity projects and infrastructures that make up the global network of ongoing efforts in the domain, and in the context of ELIXIR’s participation and contribution to the network, the following two examples highlight ongoing activities in the field of biodiversity and in the context of the European research sphere.

### Example: Biodiversity Community Integrated Knowledge Library (BiCIKL)

Several ELIXIR Nodes are involved in European projects with a focus on biodiversity. The BiCIKL project is building the Biodiversity Knowledge Hub (BKH) - a single knowledge portal to interlinked machine-readable FAIR data - using unique stable identifiers on specimens, sequences, taxonomy and publications ( Penev *et al.*, 2021, 2022). A set of core global biodiversity databases (GBIF, ENA, PlutoF, Plazi, DiSSCo, OpenBioDiv, ToL, *etc.*) are contributing with the aim to develop services to augment the interlinking of biodiversity contents, starting with biotic interactions. The project is also financing competitive implementation studies to develop transnational resources.

### Example: European Open Science Cloud (EOSC)

The European Open Science Cloud initiative (2023) intends to offer a federated and open multi-disciplinary environment where tools, data and services can be published, sought, and re-used. Via enabling seamless access and FAIR management EOSC aims to develop a Web of FAIR Data and services for science, innovation and education in Europe through which value-added services can be offered. The EOSC-Life initiative connects 13 life science ‘ESFRI’ research infrastructures to create an open, digital and collaborative space for biological and medical research. Among the EOSC-Life “FAIR” published data and catalogued services (by participating RIs), ones related to biodiversity are included. The workflow for marine Genomic Observatories data analysis is such an example ( EBI, 2021).

## Conclusions: A roadmap for the ELIXIR Biodiversity Community

Considering the context discussed above in terms of the complex landscape of ongoing initiatives working to meet the most pressing needs supporting biodiversity research and services, the ELIXIR Biodiversity Community aims to contribute towards the global aim of tackling the biodiversity crisis by helping to make possible a future where:

• Large-scale sustainable data production services are meeting the routine needs of hundreds of laboratories and thousands of citizen scientists for sequence-based biodiversity research and biomonitoring;
• ELIXIR is part of a network of well-connected, stable, and long-term infrastructures that is supporting a growing portfolio of stakeholders in biodiversity research by improving their access to, and integration of well-curated, high-quality, richly annotated, and connected molecular data.
• State-of-the-art computational tools and services are available for large-scale projects related to biodiversity, including data standardisation initiatives, allowing for the enhanced exploitation of the collected and connected data within the biodiversity knowledge graph.

To define a roadmap for the ELIXIR Biodiversity Community to help drive progress towards advances in these three overarching priority areas – data production, data integration, and data exploitation – a set of five guiding goals has been established:

1. To enhance ELIXIR’s network of networks in helping to deliver connected data for biodiversity research by: exposing and augmenting relevant ELIXIR services and infrastructures contributing to efforts to catalogue, protect, and restore biodiversity and ecosystem services; supporting ELIXIR Nodes in expanding their activities related to biodiversity data and research and relevant for the new programme priority areas; and communicating ELIXIR activities and services relevant to the biodiversity domain to broader audiences including scientists, policy makers, and the general public.
2. To support the development of standards and promote best practices in biodiversity research by: supporting and promoting the development and use of global standards, formats, guidelines, and ontologies across the domain; supporting the development of best practices in Research Data Management for biodiversity research, with a focus on molecular data but not excluding other areas; and collecting, exposing, and maintaining, a dedicated RDMkit domain for biodiversity collating relevant documentation and tools that support good practices in research data management.
3. To promote tools and workflows that facilitate reliable and reproducible biodiversity data analyses by: identifying, curating, and promoting high-quality biodiversity-related analysis tools and services to the wider community of users; connecting developers with data-generation initiatives and data users to fuel synergies that deliver tools and workflows serving their needs; and maintaining curated catalogues of FAIR biodiversity-focused tools and workflows in Bio.tools and WorkflowHub.
4. To enhance biodiversity database/infrastructure usability and interoperability by: identifying and acting on opportunities to develop technical solutions that improve connectivity amongst heterogenous biodiversity data infrastructures and resources; fostering collaborative exchanges between users and providers of biodiversity-related databases/infrastructures to improve usability and functionality; and promoting the usage of persistent identifiers across the domain, including taxonomies as the backbone of biodiversity knowledge.
5. To foster knowledge transfer in biodiversity data management and analysis by: supporting community-driven skills sharing focused on understanding how to benefit from the use of available standards and best practices; connecting developers of tools/workflows/databases with user communities through training that responds to changing technologies and associated services; and exposing collections of training materials, for example through TeSS, the Galaxy Training Network, and RDMkit.

To complement these guiding goals, the ELIXIR Biodiversity Community proposes a roadmap towards achieving our long-term objectives. Table 3 shows five long-term objectives for the ELIXIR Biodiversity Community to address, centred on engaging with stakeholders, aligning infrastructures, contributing to policy, supporting production, and enhancing ELIXIR’s portfolio in the biodiversity domain. The current focus is on the informatics, databases, and tools more than on the biological questions, so as the Community grows, it will be important to widen the diversity of its membership to ensure that the technical developments will serve the needs of biodiversity researchers. Beyond the ELIXIR Biodiversity Community itself, it is also vital to engage with other communities in the domain, including with stakeholders such as practitioners and citizen science initiatives in order to contribute towards bridging the gaps between research and implementation ( Dubois *et al.*, 2020; Fraisl *et al.*, 2022).

**Table 3.** The ELIXIR Biodiversity Community long-term objectives.

Objectives	Example approaches
Identify and support key stakeholders in the biodiversity domain	• Support efforts to harmonise data management activities within large-scale projects and transcending initiatives to result in high-quality, interoperable data and metadata • Build routes for user communities to access and add to the knowledge graph (curation) of growing resources *e.g.*, trait measurements, observations beyond geolocations *etc.* • Include primary production sectors (industry *etc.*) affecting biodiversity in the collaborative processes aiming to identify data-informed win-win solutions
Connect and align biodiversity infrastructures	• Foster interactions and promote the alignment of key infrastructures contributing to worldwide efforts to sequence and catalogue Earth’s biodiversity • Increase interoperability in biodiversity infrastructures through alignment of taxonomies and data/metadata standards • Work towards the inclusion of relevant citizen science initiatives ( *e.g.*, Atlas of Living Australia, iNaturalist, eBird) in the biodiversity infrastructure landscape
Contribute to data-informed policy decision making	• Facilitate high-level alignment of strategy and policy in the biodiversity data domain • Support reconciliation of the interests of primary producers in biodiversity-rich environments
Deliver production services for sequence-based biodiversity monitoring	• Identify and address gaps in the platforms/frameworks that exist to support the biodiversity data life cycle • Coordinate and integrate services that support workflows through all stages of the process: from sampling, taxonomic identification and vouchering, sequence generation, annotation, cataloguing and further application of the data • Reinforce the network of services that meet the route needed by hundreds of labs and thousands of citizen scientists
Connect to and leverage the full potential of ELIXIR	• Establish the network of Nodes through integrated bioinformatics resources, tools, and services delivery • Leverage and invest in training expertise and networks to connect user communities with developers of data science solutions • Focus on community integration and re-use (rather than disjointed efforts) to exploit ELIXIR tools and services that can support the biodiversity data ecosystem • Connect with other ongoing ELIXIR efforts in data production and management, standards and best practices development, as well as in analysis and exploitation

## Data Availability

No data are associated with this article. Figshare: Extended Data 1: Biodiversity RIs & Projects. https://doi.org/10.6084/m9.figshare.22723432 ( Waterhouse, 2023). This project contains the following extended data:
-
Extended_Data_1_Biodiversity_RIs_Projects.xlsx (A non-exhaustive list of biodiversity research infrastructures, collected as part of the development of the ELIXIR Biodiversity Community white paper 2022-2023.) Data are available under the terms of the Creative Commons Zero “No rights reserved” data waiver (CC0 1.0 Public domain dedication).

## References

[ref1] Abarenkov K : PlutoF—a Web Based Workbench for Ecological and Taxonomic Research, with an Online Implementation for Fungal ITS Sequences. *Evol. Bioinforma.* 2010;6:EBO.S6271. 10.4137/EBO.S6271

[ref2] Balech B Sandionigi A Marzano M : MetaCOXI: an integrated collection of metazoan mitochondrial cytochrome oxidase subunit-I DNA sequences. Database. 2022:baab084. 2022. 10.1093/database/baab084 PMC9216479 35134858

[ref3] BGE: BGE - Biodiversity Genomics Europe. 2023. (Accessed April 7, 2023). Reference Source

[ref4] Bispo A Willenz P Hajdu E : Diving into the unknown: fourteen new species of haplosclerid sponges (Demospongiae: Haplosclerida) revealed along the Peruvian coast (Southeastern Pacific). *Zootaxa.* 2022;5087:201–252. 10.11646/zootaxa.5087.2.1 35390918

[ref5] Black M EDAM: the bioscientific data analysis ontology (update 2021). 2022. 10.7490/F1000RESEARCH.1118900.1

[ref58] Blaxter M Floyd R : Molecular taxonomics for biodiversity surveys: already a reality. *Trends Ecol. Evol.* 2003;18:268–269. 10.1016/S0169-5347(03)00102-2

[ref6] Blicharska M : Biodiversity’s contributions to sustainable development. *Nat. Sustain.* 2019;2:1083–1093. 10.1038/s41893-019-0417-9

[ref7] Boekhout T : Trends in yeast diversity discovery. *Fungal Divers.* 2022;114:491–537. 10.1007/s13225-021-00494-6

[ref59] Breed MF : The potential of genomics for restoring ecosystems and biodiversity. *Nat. Rev. Genet.* 2019;20:615–628. 10.1038/s41576-019-0152-0 31300751

[ref8] Capella-Gutierrez S : Lessons Learned: Recommendations for Establishing Critical Periodic Scientific Benchmarking. *Bioinformatics.* 2017. 10.1101/181677

[ref9] Chimeno C : Peering into the Darkness: DNA Barcoding Reveals Surprisingly High Diversity of Unknown Species of Diptera (Insecta) in Germany. *Insects.* 2022;13:82. 10.3390/insects13010082 35055925 PMC8779287

[ref10] Costello MJ May RM Stork NE : Can We Name Earth’s Species Before They Go Extinct? *Science.* 2013;339:413–416. 10.1126/science.1230318 23349283

[ref78] Dubois NS Gomez A Carlson S : Bridging the research-implementation gap requires engagement from practitioners. *Conserv. Sci. Pract.* 2020;2: e134. 10.1111/csp2.134

[ref11] EBI: A workflow for marine genomic data analysis. 2021. (Accessed April 7, 2023). Reference Source

[ref12] ELIXIR: ELIXIR|ESFRI Roadmap 2021. 2021. (Accessed April 7, 2023). Reference Source

[ref57] EOSC Portal: EOSC Portal. 2023. (Accessed April 7, 2023). Reference Source

[ref13] ERGA: The European Reference Genome Atlas (ERGA) initiative. erga. 2023. (Accessed April 7, 2023). Reference Source

[ref14] ESFRI: ESFRI Strategy Report and Roadmap 2018. 2018. (Accessed April 7, 2023). Reference Source

[ref15] ESFRI: ESFRI Strategy Report on Research Infrastructures. 2021. (Accessed April 7, 2023). Reference Source

[ref16] FAO: *The state of the world’s biodiversity for food and agriculture.* Rome: FAO Commission on Genetic Resources for Food and Agriculture;2019.

[ref60] Finn C Grattarola F Pincheira-Donoso D : More losers than winners: investigating Anthropocene defaunation through the diversity of population trends. *Biol. Rev.* 2023;98:1732–1748. 10.1111/brv.12974 37189305

[ref17] Formenti G : The era of reference genomes in conservation genomics. *Trends Ecol. Evol.* 2022;37:197–202. 10.1016/j.tree.2021.11.008 35086739 PMC13065249

[ref79] Fraisl D Hager G Bedessem B : Citizen science in environmental and ecological sciences. *Nat. Rev. Methods Primer.* 2022;2:64. 10.1038/s43586-022-00144-4

[ref18] Frumkin H Haines A : Global Environmental Change and Noncommunicable Disease Risks. *Annu. Rev. Public Health.* 2019;40:261–282. 10.1146/annurev-publhealth-040218-043706 30633714

[ref19] Gadelha LMR : A survey of biodiversity informatics: Concepts, practices, and challenges. *WIREs Data Min. Knowl. Discov.* 2021;11. 10.1002/widm.1394

[ref20] GBF: Kunming-Montreal Global Biodiversity Framework. 2023. (Accessed April 7, 2023). Reference Source

[ref21] Goble C : Implementing FAIR Digital Objects in the EOSC-Life Workflow Collaboratory. 2021. 10.5281/ZENODO.4605654

[ref22] Harrow J : ELIXIR-EXCELERATE: establishing Europe’s data infrastructure for the life science research of the future. *EMBO J.* 2021;40:e107409. 10.15252/embj.2020107409 33565128 PMC7957415

[ref61] Heil KF Garrard C : *ELIXIR Communities Handbook 2024.* 2024; 10.7490/F1000RESEARCH.1119695.1

[ref23] Hermoso V : The EU Biodiversity Strategy for 2030: Opportunities and challenges on the path towards biodiversity recovery. *Environ. Sci. Policy.* 2022;127:263–271. 10.1016/j.envsci.2021.10.028

[ref24] Hoban S : Genetic diversity targets and indicators in the CBD post-2020 Global Biodiversity Framework must be improved. *Biol. Conserv.* 2020;248:108654. 10.1016/j.biocon.2020.108654

[ref25] Hoban S : Global Commitments to Conserving and Monitoring Genetic Diversity Are Now Necessary and Feasible. *Bioscience.* 2021;71:964–976. 10.1093/biosci/biab054 34475806 PMC8407967

[ref26] Hobern D : BIOSCAN: DNA barcoding to accelerate taxonomy and biogeography for conservation and sustainability Adamowicz, S, editor. *Genome.* 2021;64:161–164. 10.1139/gen-2020-0009 32268069

[ref62] Huang X : Willing or unwilling to share primary biodiversity data: results and implications of an international survey. *Conserv. Lett.* 2012;5:399–406. 10.1111/j.1755-263X.2012.00259.x

[ref27] IPBES: Global assessment report on biodiversity and ecosystem services of the Intergovernmental Science-Policy Platform on Biodiversity and Ecosystem Services. *Zenodo.* 2019. 10.5281/ZENODO.3831673

[ref28] IPCC: *Climate Change 2022: Impacts, Adaptation, and Vulnerability. Contribution of Working Group II to the Sixth Assessment Report of the Intergovernmental Panel on Climate Change.* Cambridge, UK and New York, NY, USA: Cambridge University Press;2022. Reference Source

[ref29] Ison J : The bio.tools registry of software tools and data resources for the life sciences. *Genome Biol.* 2019;20:164. 10.1186/s13059-019-1772-6 31405382 PMC6691543

[ref30] Jetz W : Essential biodiversity variables for mapping and monitoring species populations. *Nat. Ecol. Evol.* 2019;3:539–551. 10.1038/s41559-019-0826-1 30858594

[ref31] Jiménez RC : Four simple recommendations to encourage best practices in research software. *F1000Res.* 2017;6:876. 10.12688/f1000research.11407.1 28751965 PMC5490478

[ref32] Johnson CN : Biodiversity losses and conservation responses in the Anthropocene. *Science.* 2017;356:270–275. 10.1126/science.aam9317 28428393

[ref63] Karp A : Molecular technologies for biodiversity evaluation: Opportunities and challenges. *Nat. Biotechnol.* 1997;15:625–628. 10.1038/nbt0797-625 9219262

[ref33] Kissling WD : Building essential biodiversity variables (EBVs) of species distribution and abundance at a global scale. *Biol. Rev.* 2018;93:600–625. 10.1111/brv.12359 28766908

[ref77] König C Weigelt P Schrader J : Biodiversity data integration—the significance of data resolution and domain Mace, GM, editor. *PLOS Biol.* 2019;17: e3000183. 10.1371/journal.pbio.3000183 30883539 PMC6445469

[ref64] Lannom L Koureas D Hardisty AR : FAIR Data and Services in Biodiversity Science and Geoscience. *Data Intell.* 2020;2:122–130. 10.1162/dint_a_00034

[ref65] Levin N : Biodiversity data requirements for systematic conservation planning in the Mediterranean Sea. *Mar. Ecol. Prog. Ser.* 2014;508:261–281. 10.3354/meps10857

[ref34] Lewin HA : Earth BioGenome Project: Sequencing life for the future of life. *Proc. Natl. Acad. Sci.* 2018;115:4325–4333. 10.1073/pnas.1720115115 29686065 PMC5924910

[ref35] Lewin HA : The Earth BioGenome Project 2020: Starting the clock. *Proc. Natl. Acad. Sci.* 2022;119:e2115635118. 10.1073/pnas.2115635118 35042800 PMC8795548

[ref36] Marselle MR : Pathways linking biodiversity to human health: A conceptual framework. *Environ. Int.* 2021;150:106420. 10.1016/j.envint.2021.106420 33556912

[ref66] Mazzoni CJ Ciofi C Waterhouse RM : Biodiversity: an atlas of European reference genomes. *Nature.* 2023;619:252. 10.1038/d41586-023-02229-w 37433931

[ref67] McGee KM Robinson CV Hajibabaei M : Gaps in DNA-Based Biomonitoring Across the Globe. *Front. Ecol. Evol.* 2019;7:337. 10.3389/fevo.2019.00337

[ref37] Mora C Tittensor DP Adl S : How Many Species Are There on Earth and in the Ocean? Mace, GM, editor. *PLoS Biol.* 2011;9:e1001127. 10.1371/journal.pbio.1001127 21886479 PMC3160336

[ref38] Moura MR Jetz W : Shortfalls and opportunities in terrestrial vertebrate species discovery. *Nat. Ecol. Evol.* 2021;5:631–639. 10.1038/s41559-021-01411-5 33753900

[ref39] Nature. : Biodiversity faces its make-or-break year, and research will be key. *Nature.* 2022;601:298–298. 10.1038/d41586-022-00110-w 35042999

[ref40] Nic Lughadha E : Extinction risk and threats to plants and fungi. *PLANTS PEOPLE PLANET.* 2020;2:389–408. 10.1002/ppp3.10146

[ref41] Ommen KE van : ARISE: Building an infrastructure for species recognition and biodiversity monitoring in the Netherlands. *Biodivers. Inf. Sci. Stand.* 2022;6:e93613. 10.3897/biss.6.93613

[ref42] Page R : Towards a biodiversity knowledge graph. *Res. Ideas Outcomes.* 2016;2:e8767. 10.3897/rio.2.e8767

[ref43] Page RDM : BioNames: linking taxonomy, texts, and trees. *PeerJ.* 2013;1:e190. 10.7717/peerj.190 24244913 PMC3817598

[ref68] Pearman PB : Monitoring of species’ genetic diversity in Europe varies greatly and overlooks potential climate change impacts. *Nat. Ecol. Evol.* 2024;8:267–281. 10.1038/s41559-023-02260-0 38225425 PMC10857941

[ref44] Penev L : Towards Interlinked FAIR Biodiversity Knowledge: The BiCIKL perspective. *Biodivers. Inf. Sci. Stand.* 2021;5:e74233. 10.3897/biss.5.74233

[ref70] Penev L : Biodiversity Community Integrated Knowledge Library (BiCIKL). *Res. Ideas Outcomes.* 2022;8: e81136. 10.3897/rio.8.e81136

[ref71] Pereira HM Navarro LM Martins IS : Global Biodiversity Change: The Bad, the Good, and the Unknown. *Annu. Rev. Environ. Resour.* 2012;37:25–50. 10.1146/annurev-environ-042911-093511

[ref72] Porter TM Hajibabaei M : Scaling up: A guide to high-throughput genomic approaches for biodiversity analysis. *Mol. Ecol.* 2018;27:313–338. 10.1111/mec.14478 29292539

[ref45] Rahman MM Burian A Creedy TJ : DNA -based assessment of environmental degradation in an unknown fauna: The freshwater macroinvertebrates of the Indo-Burmese hotspot. *J. Appl. Ecol.* 2022;59:1644–1658. 10.1111/1365-2664.14174

[ref46] Robinson WD Peres CA : Editorial: Benchmarking Biodiversity in an Era of Rapid Change. *Front. Ecol. Evol.* 2021;9:810287. 10.3389/fevo.2021.810287

[ref47] Segelbacher G : New developments in the field of genomic technologies and their relevance to conservation management. *Conserv. Genet.* 2022;23:217–242. 10.1007/s10592-021-01415-5

[ref48] Smith V : Research Infrastructure Contact Zones: a framework and dataset to characterise the activities of major biodiversity informatics initiatives. *Biodiversity Data Journal.* 2022;10. 10.3897/arphapreprints.e82955 36761622 PMC9848541

[ref73] Soulé ME Wilcox BA , editors: *Conservation biology: an evolutionary-ecological perspective.* Sunderland, Mass: Sinauer Associates;1980.

[ref49] Theissinger K : How genomics can help biodiversity conservation. *Trends Genet.* 2023. S0168952523000203. 10.1016/j.tig.2023.01.005 36801111

[ref50] Turvey ST Crees JJ : Extinction in the Anthropocene. *Curr. Biol.* 2019;29:R982–R986. 10.1016/j.cub.2019.07.040 31593681

[ref51] UN: THE 17 GOALS|Sustainable Development. 2015. (Accessed April 7, 2023). Reference Source

[ref52] Veiga Leprevost F da : BioContainers: an open-source and community-driven framework for software standardization Valencia, A, editor. *Bioinformatics.* 2017;33:2580–2582. 10.1093/bioinformatics/btx192 28379341 PMC5870671

[ref53] Waterhouse RM : Recommendations for connecting molecular sequence and biodiversity research infrastructures through ELIXIR. *F1000Res.* 2022;10:1238. 10.12688/f1000research.73825.2 35999898 PMC9360911

[ref54] Waterhouse RM : Extended Data 1: Biodiversity RIs & Projects.[Dataset]. *figshare.* 2023. 10.6084/m9.figshare.22723432

[ref74] Waterhouse R Burgin J Gabaldón T : *The ELIXIR biodiversity community: Actions, goals, & implementation study.* 2023. 10.7490/F1000RESEARCH.1119439.1

[ref75] Wetzel FT : Unlocking biodiversity data: Prioritization and filling the gaps in biodiversity observation data in Europe. *Biol. Conserv.* 2018;221:78–85. 10.1016/j.biocon.2017.12.024

[ref55] Wezel A : Agroecological principles and elements and their implications for transitioning to sustainable food systems. A review. *Agron. Sustain. Dev.* 2020;40:40. 10.1007/s13593-020-00646-z

[ref76] Wilkinson MD : The FAIR Guiding Principles for scientific data management and stewardship. *Sci. Data.* 2016;3: 160018. 10.1038/sdata.2016.18 26978244 PMC4792175

[ref56] WWF: *The Living Planet Report 2022 – Building a nature-positive society.* Almond REA Grooten M Juffe Bignoli D , editors. WWF; Reference Source 2022.

