# Supplementary material for: The ELIXIR Biodiversity Community: Understanding short- and long-term changes in biodiversity
Source: F1000Res. 2024 May 22;12:ELIXIR-499. Originally published 2023 May 15. [Version 2] doi: 10.12688/f1000research.133724.2 (PMC11179050; doi:10.12688/f1000research.133724.2)
Supplement: Peer review history

# Reviewer report: Reviewer response for version 2

**DOI:** 10.5256/f1000research.166159.r281457

**Published:** 2024-06-29

**Authors:** Abigail Benson

**Affiliations:** 1 US Geological Survey, Colorado, USA

The author revisions sufficiently addressed the concerned raised.

Is the topic of the opinion article discussed accurately in the context of the current literature?

Partly

Are arguments sufficiently supported by evidence from the published literature?

Partly

Are all factual statements correct and adequately supported by citations?

Partly

Are the conclusions drawn balanced and justified on the basis of the presented arguments?

Partly

Reviewer Expertise:

biodiversity informatics, open science, data standards

I confirm that I have read this submission and believe that I have an appropriate level of expertise to confirm that it is of an acceptable scientific standard.

# Reviewer report: Reviewer response for version 2

**DOI:** 10.5256/f1000research.166159.r281455

**Published:** 2024-06-14

**Authors:** Cher FY Chow

**Affiliations:** 1 Centre for Biological Diversity, University of St Andrews, St Andrews, Scotland, UK

The authors have sufficiently addressed my concerns and this article can be approved.

Is the topic of the opinion article discussed accurately in the context of the current literature?

Yes

Are arguments sufficiently supported by evidence from the published literature?

Partly

Are all factual statements correct and adequately supported by citations?

No

Are the conclusions drawn balanced and justified on the basis of the presented arguments?

Yes

Reviewer Expertise:

macroecology, biodiversity data, ecoinformatics, marine ecology

I confirm that I have read this submission and believe that I have an appropriate level of expertise to confirm that it is of an acceptable scientific standard.

# Reviewer report: Reviewer response for version 2

**DOI:** 10.5256/f1000research.166159.r281456

**Published:** 2024-05-28

**Authors:** Patrick Comer

**Affiliations:** 1 NatureServe, Arlington, Virginia, USA

I think the paper is fine in its revised form.

Is the topic of the opinion article discussed accurately in the context of the current literature?

Yes

Are arguments sufficiently supported by evidence from the published literature?

Yes

Are all factual statements correct and adequately supported by citations?

Yes

Are the conclusions drawn balanced and justified on the basis of the presented arguments?

Partly

Reviewer Expertise:

Biodiversity Conservation, Landscape Ecology, Biodiversity status assessment, Climate change vulnerability and adaptation

I confirm that I have read this submission and believe that I have an appropriate level of expertise to confirm that it is of an acceptable scientific standard.

# Reviewer report: Reviewer response for version 1

**DOI:** 10.5256/f1000research.146735.r232449

**Published:** 2024-03-05

**Authors:** Cher FY Chow

**Affiliations:** 1 Centre for Biological Diversity, University of St Andrews, St Andrews, Scotland, UK

Waterhouse et al. present here an opinion article presenting ELIXIR as a solution that can help standardise biodiversity monitoring at scale for the European region and globally. This paper first describes the current landscape of challenges and responses to biodiversity monitoring, and later presents the efforts undertaken by the ELIXIR consortium that can address this. There is a demonstrated need to detect biodiversity change in a coordinated manner at a large spatial scale as emphasised by priorities set by IPBES, Global Biodiversity Framework, and the UN Sustainable Goals. The authors outline the current needs in biodiversity research and policy efforts and highlight the utility of incorporating more “omics” and molecular techniques. They discuss a complex landscape of biodiversity informatics, where challenges exist from data collection to data management and linkages across sources. They present the ELIXIR consortium infrastructure and network of projects/initiatives which help address these challenges.

 I first would like to commend the authors for their effort in tackling a complex set of challenges. I appreciate the practical nature of this article, and I can envision it targeted as a relevant reference for European governmental bodies and researchers. However, the article currently needs further development in order to demonstrate to readers 1) the authors’ concept of biodiversity informatics, 2) where in this network there are gaps or challenges, and finally 3) how its breadth of initiatives, tools, and platforms can be used by scientists or environmental managers. I outline below my general concerns and questions I would like the authors to respond to.

**Article aim: **I understand this article is currently presented as an opinion article. The authors state their aim the in the abstract as “[to present] a set of plans, both technical and community-oriented, that should both enhance how ELIXIR Services are applied in the biodiversity field and how ELIXIR builds connections across the many other infrastructures active in this area.” However, from my reading, it is unclear to me what questions or knowledge gaps the authors are tackling, or presented opinions, and secondly, what the authors want me to take away from the article. Are they presenting the results from the ELIXIR consortium? If the aim is to provide some roadmap of resources within biodiversity informatics, I find the latter half of the article difficult to interpret and apply from a user perspective. It is unclear to me how some projects listed in Table 1, e.g. a barcoding project based in the Norfolk Broads on page 6, meets informatics or infrastructure challenges outlined previously. This needs to be made clearer and more explicit to the reader.

 Furthermore, I find the title a mismatch from the article contents and would suggest the authors adjust it to better reflect the aims.

**Introduction of ELIXIR: **Please include a section that introduces ELIXIR and its structure. As it is presented, the consortium is mentioned and introduced in parts interspersed throughout the first five pages but not completely.

**Biodiversity change and molecular approaches: **The authors introduce biodiversity change, global policy responses, and the need for incorporating molecular techniques. However, the authors have not demonstrated a clear conceptual link between molecular approaches and the crisis of biodiversity loss and extinction. There are several facets to biodiversity and change is occurring in a myriad of patterns and scales (e.g. [1]; [2] ; [3]; [4]; [5]. Genetic diversity assessments, enhancing species detections through eDNA, and resolved taxonomy are examples of critical components to understanding biodiversity change. The authors hint at these but not in a clear, demonstrated manner. How is a lack of high-throughput and -omics approaches failing our understanding of biodiversity? How can genetic diversity data be linked with the research examples listed under “Scientific opportunities in biodiversity research”? Are there examples where this potential is demonstrated?

**The network of biodiversity knowledge: **I like Figure 1 as a conceptual figure to ground the idea of how biodiversity monitoring or research requires a network of components. From my knowledge of biodiversity informatics, this concept forms the basis of the first five pages. However, the authors should clearly state this conceptual landscape in the introduction before presenting challenges. This can then demonstrate to the reader how molecular approaches fit into that framework.

**Citations needed:**

 Page 5 can be improved by engaging and contextualising its ideas with existing literature on biodiversity informatics and data management. The ELIXIR consortium is not alone in researching how to improve infrastructure for biodiversity data. The ideas presented in this article build upon previous work. This should be recognised. For example:

- Page 4 under “Biodiversity research and infrastructures increasingly rely on molecular data”: [12];[13]; [7]
- Page 5: “FAIRification of digital research objects… is increasingly recognised as essential in biodiversity research”: [8]; [9] 
- Page 5: “This heterogeneity, however, gives rise to many challenges, both technical in terms of data analysis (due to inadequacies of existing methodologies), data integration and data interaction, and at the level of the scientific community, which faces a heterogeneous landscape of infrastructures and resources that can be difficult to navigate.”: [11]; [12]; [10]

**Minor comments**

 I appreciate that ELIXIR is an extensive research infrastructure project and have been well-represented in other published works. This paper should stand alone without relying on previously published works for introduction. There are several instances of unintroduced abbreviations which assumes that the reader is already familiar with the organisations involved. Please include clearer introductions of ELIXIR as a consortium and mentioned nodes.

Is the topic of the opinion article discussed accurately in the context of the current literature?

Yes

Are arguments sufficiently supported by evidence from the published literature?

Partly

Are all factual statements correct and adequately supported by citations?

No

Are the conclusions drawn balanced and justified on the basis of the presented arguments?

Yes

Reviewer Expertise:

macroecology, biodiversity data, ecoinformatics, marine ecology

I confirm that I have read this submission and believe that I have an appropriate level of expertise to state that I do not consider it to be of an acceptable scientific standard, for reasons outlined above.

## References

[1] : Global Biodiversity Change: The Bad, the Good, and the Unknown. *Annual Review of Environment and Resources* .2012;37(1) : 10.1146/annurev-environ-042911-093511 25-50 10.1146/annurev-environ-042911-093511

[2] : Estimates of local biodiversity change over time stand up to scrutiny. *Ecology* .2017;98(2) : 10.1002/ecy.1660 583-590 10.1002/ecy.1660 27864922

[3] : Clustered versus catastrophic global vertebrate declines. *Nature* .2020;588(7837) : 10.1038/s41586-020-2920-6 267-271 10.1038/s41586-020-2920-6 33208939

[4] : A millennium of increasing diversity of ecosystems until the mid-20th century. *Glob Chang Biol* .2022;28(20) : 10.1111/gcb.16335 5945-5955 10.1111/gcb.16335 35808866 PMC9543278

[5] : More losers than winners: investigating Anthropocene defaunation through the diversity of population trends. *Biol Rev Camb Philos Soc* .2023;98(5) : 10.1111/brv.12974 1732-1748 10.1111/brv.12974 37189305

[6] : Scaling up: A guide to high-throughput genomic approaches for biodiversity analysis. *Mol Ecol* .2018;27(2) : 10.1111/mec.14478 313-338 10.1111/mec.14478 29292539

[7] : The FAIR Guiding Principles for scientific data management and stewardship. *Sci Data* .2016;3: 10.1038/sdata.2016.18 160018 10.1038/sdata.2016.18 26978244 PMC4792175

[8] : FAIR Data and Services in Biodiversity Science and Geoscience. *Data Intelligence* .2020;2(1-2) : 10.1162/dint_a_00034 122-130 10.1162/dint_a_00034

[9] : Unlocking biodiversity data: Prioritization and filling the gaps in biodiversity observation data in Europe. *Biological Conservation* .2018;221: 10.1016/j.biocon.2017.12.024 78-85 10.1016/j.biocon.2017.12.024

[10] : Willing or unwilling to share primary biodiversity data: results and implications of an international survey. *Conservation Letters* .2012;5(5) : 10.1111/j.1755-263X.2012.00259.x 399-406 10.1111/j.1755-263X.2012.00259.x

[11] : Biodiversity data requirements for systematic conservation planning in the Mediterranean Sea. *Marine Ecology Progress Series* .2014;508: 10.3354/meps10857 261-281 10.3354/meps10857

[12] : Molecular taxonomics for biodiversity surveys: already a reality. *Trends in Ecology & Evolution* .2003;18(6) : 10.1016/S0169-5347(03)00102-2 268-269 10.1016/S0169-5347(03)00102-2

[13] : Molecular technologies for biodiversity evaluation: opportunities and challenges. *Nat Biotechnol* .1997;15(7) : 10.1038/nbt0797-625 625-8 10.1038/nbt0797-625 9219262

# Response

**Published:** 2024-05-07

**Authors:** Robert Waterhouse

**Affiliations:** University of Geneva, Switzerland

Waterhouse et al. present here an opinion article presenting ELIXIR as a solution that can help standardise biodiversity monitoring at scale for the European region and globally. This paper first describes the current landscape of challenges and responses to biodiversity monitoring, and later presents the efforts undertaken by the ELIXIR consortium that can address this. There is a demonstrated need to detect biodiversity change in a coordinated manner at a large spatial scale as emphasised by priorities set by IPBES, Global Biodiversity Framework, and the UN Sustainable Goals. The authors outline the current needs in biodiversity research and policy efforts and highlight the utility of incorporating more “omics” and molecular techniques. They discuss a complex landscape of biodiversity informatics, where challenges exist from data collection to data management and linkages across sources. They present the ELIXIR consortium infrastructure and network of projects/initiatives which help address these challenges.

**RESPONSE ⇒** We thank the reviewer for their positive summary.

 I first would like to commend the authors for their effort in tackling a complex set of challenges. I appreciate the practical nature of this article, and I can envision it targeted as a relevant reference for European governmental bodies and researchers. However, the article currently needs further development in order to demonstrate to readers 1) the authors’ concept of biodiversity informatics, 2) where in this network there are gaps or challenges, and finally 3) how its breadth of initiatives, tools, and platforms can be used by scientists or environmental managers. I outline below my general concerns and questions I would like the authors to respond to.

**RESPONSE ⇒** We thank the reviewer for their appreciation of our efforts to describe the goals of the Community and how they fit in the broader landscape. The section in the introduction “Informatics challenges facing biodiversity infrastructures and resources” was intended to briefly summarise the current landscape of biodiversity informatics, pointing to a previous output of the ELIXIR Biodiversity Community - “Recommendations for connecting molecular sequence and biodiversity research infrastructures through ELIXIR” (Waterhouse et al, 2022) - where we covered this topic in much more detail, including outlining the gaps and challenges. We have now summarised these primary “needs” as emerging themes from our prior survey (included in the much extended section ‘The ELIXIR Biodiversity Community: An “ecosystem” of projects’. We also summarised the activities of the Community Implementation Study, which exemplifies how ELIXIR tools and services are being developed and connected for use by researchers.

 ADDED ⇒ ELIXIR Communities are groups of experts across ELIXIR Nodes and beyond that represent a scientific or technological theme which drives the development of standards, services, and/or training in and across services offered by ELIXIR, thereby connecting the infrastructure services to research domains (Heil & Garrard, 2024). The ELIXIR Biodiversity Community was first launched in 2019 as a Focus Group to develop and coordinate ELIXIR Nodes’ tools, resources, and research work connected to the biodiversity domain. As part of the process of maturing from a Focus Group to a Community, members initiated activities including: (1) cataloguing ELIXIR Services that support biodiversity research; (2) developing and publishing their “Recommendations for connecting molecular sequence and biodiversity research infrastructures through ELIXIR” (Waterhouse et al., 2022); (3) coordinating ELIXIR Node participation in Horizon Europe project proposals - The Biodiversity Community Integrated Knowledge Library (BiCIKL) and Biodiversity Genomics Europe (BGE); and (4) beginning to establish connections with key external partners/projects in the biodiversity domain (such as those listed in Table 1); leading to the formal recognition in 2022 as an ELIXIR Community (Waterhouse et al., 2023). 

 ADDED ⇒ Operationally, monthly online meetings coordinated by the Community co-leads with support from the ELIXIR Hub serve as the primary forum for interactions, complemented by discussions and notifications on the ELIXIR Slack Workspace’s Biodiversity Community channel. These include sharing information on members’ participation in ongoing or planned biodiversity-related projects and initiatives, including the Community-led Implementation Study “Biodiversity Networks for ELIXIR”. The online meetings also feature presentations on tools and services developed by ELIXIR Nodes as well as hosting invited speakers representing key external partners/projects. The Community’s Implementation Study encompasses four key areas of work to drive Community activities: (1) to survey and catalogue Research Data Management (RDM) elements relevant to the biodiversity domain, with a focus on molecular data; (2) to catalogue, review, and categorise tools, services, and analytical workflows currently in use by ELIXIR Nodes and the wider community, that process and analyse biodiversity-related data; (3) to describe the landscape of stakeholders ELIXIR is working with or needs to better engage with to establish a “network of networks” for biodiversity research and services; and (4) to leverage the strengths of ELIXIR’s training experience to help support the growth of the Biodiversity Community through network-driven sharing of training experiences and knowledge transfer and materials. Together, these actions are serving to enhance ELIXIR’s network of networks in helping to deliver connected data to advance biodiversity research.

 ADDED ⇒ In terms of informatics solutions connected to such projects, the ELIXIR Biodiversity Community is guided by themes emerging from surveying approaches by which molecular technologies are helping to inform understanding of biodiversity (Waterhouse et al., 2022): biodiversity-related and informatics infrastructures need to develop close and strategic collaborations; work on taxonomy needs to be better aligned and standardised across different infrastructures and fields of study; metadata urgently needs harmonisation and common approaches to research data management must be widely adopted; current data science solutions need to be scaled up to address the rapidly accumulating amounts of molecular data; bioinformatics support for biodiversity research needs to be made widely available and properly maintained; user training on biodiversity research tools, services, and infrastructures needs to be prioritised; and community initiatives need to be collaborative, proactive, and solution-driven.

 Article aim: I understand this article is currently presented as an opinion article. The authors state their aim the in the abstract as “[to present] a set of plans, both technical and community-oriented, that should both enhance how ELIXIR Services are applied in the biodiversity field and how ELIXIR builds connections across the many other infrastructures active in this area.” However, from my reading, it is unclear to me what questions or knowledge gaps the authors are tackling, or presented opinions, and secondly, what the authors want me to take away from the article. Are they presenting the results from the ELIXIR consortium? If the aim is to provide some roadmap of resources within biodiversity informatics, I find the latter half of the article difficult to interpret and apply from a user perspective. It is unclear to me how some projects listed in Table 1, e.g. a barcoding project based in the Norfolk Broads on page 6, meets informatics or infrastructure challenges outlined previously. This needs to be made clearer and more explicit to the reader.

 Furthermore, I find the title a mismatch from the article contents and would suggest the authors adjust it to better reflect the aims.

**RESPONSE ⇒ **We thank the reviewer for their frank assessment, and apologise for some lack of clarity with respect to the purpose of the White Paper publication, which would be particularly unclear to readers who are new to ELIXIR. Much of the parts dedicated to outlining other initiatives, projects, tools, services, and connections with ELIXIR Platforms and other Communities are primarily for the benefit of ELIXIR members who review the Community’s White Paper as part of the formal process of becoming an ELIXIR Community. The aim is not to present results of ELIXIR actions but rather to outline where ELIXIR might be well-placed to contribute and how as a Community we can begin to develop initiatives in the domain of biodiversity research and services by connecting and growing ELIXIR Node activities in data science that can support the field. The substantially revised roadmap section at the end of the paper is designed to articulate  the goals and practical aspects of what the Community aims to tackle and how. We hope these changes now provide a clearer picture, especially to readers who are new to ELIXIR.

 ADDED ⇒ The revisions to this final section are rather extensive, please see the new content of “Conclusions: A roadmap for the ELIXIR Biodiversity Community” for all the details.

 Introduction of ELIXIR: Please include a section that introduces ELIXIR and its structure. As it is presented, the consortium is mentioned and introduced in parts interspersed throughout the first five pages but not completely.

**RESPONSE ⇒** We have now included a brief introduction of ELIXIR and its structure.

 ADDED (Introduction) ⇒ As a European life sciences infrastructure, ELIXIR strives to coordinate bioinformatics resources from across Europe to enable researchers to access and analyse life science data, to improve the value and impact of life science research on public health, the environment, and the economy. 

 Biodiversity change and molecular approaches: The authors introduce biodiversity change, global policy responses, and the need for incorporating molecular techniques. However, the authors have not demonstrated a clear conceptual link between molecular approaches and the crisis of biodiversity loss and extinction. There are several facets to biodiversity and change is occurring in a myriad of patterns and scales (e.g. [1]; [2] ; [3]; [4]; [5]. Genetic diversity assessments, enhancing species detections through eDNA, and resolved taxonomy are examples of critical components to understanding biodiversity change. The authors hint at these but not in a clear, demonstrated manner. How is a lack of high-throughput and -omics approaches failing our understanding of biodiversity? How can genetic diversity data be linked with the research examples listed under “Scientific opportunities in biodiversity research”? Are there examples where this potential is demonstrated?

**RESPONSE ⇒** The background in the introduction was designed to be concise and general, so we avoided detailed explanations of the different facets and how they might come together to address our understanding of biodiversity change. Instead, these more technical points are presented in an earlier output of the community, emerging from surveying approaches by which molecular technologies are helping to inform understanding of biodiversity (Waterhouse et al., 2022). We have now included a summary of these themes in the much extended section describing the ELIXIR Biodiversity Community. For the examples listed in this section without references we have now added supporting references where appropriate.

 The network of biodiversity knowledge: I like Figure 1 as a conceptual figure to ground the idea of how biodiversity monitoring or research requires a network of components. From my knowledge of biodiversity informatics, this concept forms the basis of the first five pages. However, the authors should clearly state this conceptual landscape in the introduction before presenting challenges. This can then demonstrate to the reader how molecular approaches fit into that framework.

**RESPONSE ⇒** The use of Rod Page’s Biodiversity Knowledge graph as figure 1 was intended as a highly recognisable way to emphasise how data connectivity is important and that the ELIXIR Community’s current focus on molecular data does not imply that other facets are less important. The introduction sets the scene, while the main focus is on the establishment, operations, and goals of the ELIXIR Biodiversity Community in the context of that scene. Now that we have added the operational description of the Community to more clearly describe the community structure and how it interfaces with other research infrastructures and key projects/initiatives in the biodiversity domain, and greatly elaborated the Community goals we hope it is much clearer why this figure is used here rather than in the general introduction.

 Citations needed:

 Page 5 can be improved by engaging and contextualising its ideas with existing literature on biodiversity informatics and data management. The ELIXIR consortium is not alone in researching how to improve infrastructure for biodiversity data. The ideas presented in this article build upon previous work. This should be recognised. For example:

 Page 4 under “Biodiversity research and infrastructures increasingly rely on molecular data”: [12];[13]; [7]

 Page 5: “FAIRification of digital research objects… is increasingly recognised as essential in biodiversity research”: [8]; [9] 

 Page 5: “This heterogeneity, however, gives rise to many challenges, both technical in terms of data analysis (due to inadequacies of existing methodologies), data integration and data interaction, and at the level of the scientific community, which faces a heterogeneous landscape of infrastructures and resources that can be difficult to navigate.”: [11]; [12]; [10]

**RESPONSE ⇒** We thank the reviewer to pointing us to these key references that we now use to support the statements being made.

 Minor comments

 I appreciate that ELIXIR is an extensive research infrastructure project and have been well-represented in other published works. This paper should stand alone without relying on previously published works for introduction. There are several instances of unintroduced abbreviations which assumes that the reader is already familiar with the organisations involved. Please include clearer introductions of ELIXIR as a consortium and mentioned nodes.

**RESPONSE ⇒ **We thank the reviewer for this observation that we need to bring more clarity especially for readers less familiar with ELIXIR, so we have taken measures to remedy this throughout, including a brief definition presented early in the introduction.

 ADDED (Introduction) ⇒ As a European life sciences infrastructure, ELIXIR strives to coordinate bioinformatics resources from across Europe to enable researchers to access and analyse life science data, to improve the value and impact of life science research on public health, the environment, and the economy.

# Reviewer report: Reviewer response for version 1

**DOI:** 10.5256/f1000research.146735.r223867

**Published:** 2024-02-22

**Authors:** Abigail Benson, Stephen Formel

**Affiliations:** 1 US Geological Survey, Colorado, USA

The ELIXIR Biodiversity Community: Understanding short- and long-term changes in biodiversity highlights the need for biodiversity informatics to address challenges facing our world and provides lists of projects and entities working to contribute biodiversity informatics solutions.

 We were really interested in this work and excited to read it. However, we struggled with finding the conceptual thread that tied the disparate sections together. The introduction provided an important summary about biodiversity but was missing the summarization of the current landscape of biodiversity informatics that would provide the foundation for where ELIXIR fits into the puzzle. The lists of tools are valuable but too extensive and missing the connective text that was needed to understand why these projects were being listed. One challenge we had as readers is that we are new to ELIXIR and it’s not spelled out or described in the text. We were confused about the connection between ELIXIR and the threats and challenges that were described. We were left wondering which ones ELIXIR addresses and how? We had expected based on the title to learn about the ELIXIR community, how it’s organized, how it interacts, and what makes it a community but we didn’t find that information in the paper so we were left wondering what the ELIXIR community is?

 We also felt that the text could use more supporting references. There are times when strong statements are provided but are not backed up with needed citations.

 We would suggest the authors narrow the focus in the challenges section to the ones the ELIXIR community is best poised to address and make those connections explicit in the text. We also would find it beneficial for ELIXIR to be described and more thoroughly contextualized. We also found it confusing when the paper mentions another project or RI as an official collaborator with ELIXIR, and when they merely serve as example. Perhaps replacing Figure 1 with some visual explanation of the community structure would help.

 We are hopeful that the paper can be revised.

Is the topic of the opinion article discussed accurately in the context of the current literature?

Partly

Are arguments sufficiently supported by evidence from the published literature?

Partly

Are all factual statements correct and adequately supported by citations?

Partly

Are the conclusions drawn balanced and justified on the basis of the presented arguments?

Partly

Reviewer Expertise:

biodiversity informatics, open science, data standards

We confirm that we have read this submission and believe that we have an appropriate level of expertise to state that we do not consider it to be of an acceptable scientific standard, for reasons outlined above.

# Response

**Published:** 2024-05-07

**Authors:** Robert Waterhouse

**Affiliations:** University of Geneva, Switzerland

The ELIXIR Biodiversity Community: Understanding short- and long-term changes in biodiversity highlights the need for biodiversity informatics to address challenges facing our world and provides lists of projects and entities working to contribute biodiversity informatics solutions. We were really interested in this work and excited to read it. However, we struggled with finding the conceptual thread that tied the disparate sections together. The introduction provided an important summary about biodiversity but was missing the summarization of the current landscape of biodiversity informatics that would provide the foundation for where ELIXIR fits into the puzzle. The lists of tools are valuable but too extensive and missing the connective text that was needed to understand why these projects were being listed. One challenge we had as readers is that we are new to ELIXIR and it’s not spelled out or described in the text. We were confused about the connection between ELIXIR and the threats and challenges that were described. We were left wondering which ones ELIXIR addresses and how? We had expected based on the title to learn about the ELIXIR community, how it’s organized, how it interacts, and what makes it a community but we didn’t find that information in the paper so we were left wondering what the ELIXIR community is?

**RESPONSE ⇒ **We thank the reviewers for their interest and apologise that some aspects were not covered in enough detail to provide complete answers, especially to readers who are new to ELIXIR. The section in the introduction “Informatics challenges facing biodiversity infrastructures and resources” was intended to briefly summarise the current landscape of biodiversity informatics, pointing to a previous output of the ELIXIR Biodiversity Community (Waterhouse et al, 2022) where we covered this topic in much more detail. In the introduction we outlined  global challenges and potential solutions, and only later in the text we describe  how ELIXIR might fit into these landscapes - with a focus on molecular data and their integration into the broader biodiversity knowledge graph. To clarify ELIXIR’s operational standing and Community model, we have added a detailed description to the section immediately following the introduction: ‘The ELIXIR Biodiversity Community: An “ecosystem” of projects’. The substantially revised roadmap section at the end of the paper is also designed to clarify the goals and practical aspects of what the Community aims to tackle and how. Regarding the named/listed tools, projects, platforms, and other communities - this is primarily for the benefit of ELIXIR members who review the Community’s White Paper as part of the formal process of becoming an ELIXIR Community. We hope these changes now provide a clearer picture, especially to readers who are new to ELIXIR.  

 ADDED ⇒ ELIXIR Communities are groups of experts across ELIXIR Nodes and beyond that represent a scientific or technological theme which drives the development of standards, services, and/or training in and across services offered by ELIXIR, thereby connecting the infrastructure services to research domains (Heil & Garrard, 2024). The ELIXIR Biodiversity Community was first launched in 2019 as a Focus Group to develop and coordinate ELIXIR Nodes’ tools, resources, and research work connected to the biodiversity domain. As part of the process of maturing from a Focus Group to a Community, members initiated activities including: (1) cataloguing ELIXIR Services that support biodiversity research; (2) developing and publishing their “Recommendations for connecting molecular sequence and biodiversity research infrastructures through ELIXIR” (Waterhouse et al., 2022); (3) coordinating ELIXIR Node participation in Horizon Europe project proposals - The Biodiversity Community Integrated Knowledge Library (BiCIKL) and Biodiversity Genomics Europe (BGE); and (4) beginning to establish connections with key external partners/projects in the biodiversity domain (such as those listed in Table 1); leading to the formal recognition in 2022 as an ELIXIR Community (Waterhouse et al., 2023). 

 ADDED ⇒ Operationally, monthly online meetings coordinated by the Community co-leads with support from the ELIXIR Hub serve as the primary forum for interactions, complemented by discussions and notifications on the ELIXIR Slack Workspace’s Biodiversity Community channel. These include sharing information on members’ participation in ongoing or planned biodiversity-related projects and initiatives, including the Community-led Implementation Study “Biodiversity Networks for ELIXIR”. The online meetings also feature presentations on tools and services developed by ELIXIR Nodes as well as hosting invited speakers representing key external partners/projects. The Community’s Implementation Study encompasses four key areas of work to drive Community activities: (1) to survey and catalogue Research Data Management (RDM) elements relevant to the biodiversity domain, with a focus on molecular data; (2) to catalogue, review, and categorise tools, services, and analytical workflows currently in use by ELIXIR Nodes and the wider community, that process and analyse biodiversity-related data; (3) to describe the landscape of stakeholders ELIXIR is working with or needs to better engage with to establish a “network of networks” for biodiversity research and services; and (4) to leverage the strengths of ELIXIR’s training experience to help support the growth of the Biodiversity Community through network-driven sharing of training experiences and knowledge transfer and materials. Together, these actions are serving to enhance ELIXIR’s network of networks in helping to deliver connected data to advance biodiversity research.

 ADDED ⇒ In terms of informatics solutions connected to such projects, the ELIXIR Biodiversity Community is guided by themes emerging from surveying approaches by which molecular technologies are helping to inform understanding of biodiversity (Waterhouse et al., 2022): biodiversity-related and informatics infrastructures need to develop close and strategic collaborations; work on taxonomy needs to be better aligned and standardised across different infrastructures and fields of study; metadata urgently needs harmonisation and common approaches to research data management must be widely adopted; current data science solutions need to be scaled up to address the rapidly accumulating amounts of molecular data; bioinformatics support for biodiversity research needs to be made widely available and properly maintained; user training on biodiversity research tools, services, and infrastructures needs to be prioritised; and community initiatives need to be collaborative, proactive, and solution-driven.

 We also felt that the text could use more supporting references. There are times when strong statements are provided but are not backed up with needed citations.

 RESPONSE ⇒ We have included a number of additional references in the revised manuscript. If there are additional specific recommendations we would be happy to incorporate them into the text as needed.

 We would suggest the authors narrow the focus in the challenges section to the ones the ELIXIR community is best poised to address and make those connections explicit in the text. We also would find it beneficial for ELIXIR to be described and more thoroughly contextualized. We also found it confusing when the paper mentions another project or RI as an official collaborator with ELIXIR, and when they merely serve as example. Perhaps replacing Figure 1 with some visual explanation of the community structure would help.

**RESPONSE ⇒ **We designed the introduction to first encompass the broader perspectives on key challenges in biodiversity research, including highlighting what is needed to advance the status quo, and then follow that up with “ELIXIR’s roles”, i.e. where ELIXIR activities could contribute to meeting these challenges. We have revised the text in parts to clarify the motivation to contribute to the wider landscape of efforts, bringing expertise in data management solutions, particularly with respect to molecular data. We added some context on ELIXIR as a whole (introduction), and we now describe the Community operations and key themes that guide Community activities connected to biodiversity-related projects and initiatives. The considerable re-working of the concluding roadmap section is aimed at narrowing the focus on where the ELIXIR Biodiversity Community is best poised to contribute. The use of Rod Page’s Biodiversity Knowledge graph as figure 1 emphasises why data connectivity is important and that the ELIXIR Community’s current focus on molecular data does not imply that other facets are less important. We hope that the added operational description of the Community now addresses the community structure and how it interfaces with other research infrastructures and key projects/initiatives in the biodiversity domain.

 ADDED (Introduction) ⇒ As a European life sciences infrastructure, ELIXIR strives to coordinate bioinformatics resources from across Europe to enable researchers to access and analyse life science data, to improve the value and impact of life science research on public health, the environment, and the economy.

 ADDED (‘The ELIXIR Biodiversity Community: An “ecosystem” of projects’.) ⇒ See the added text in the response above. 

 We are hopeful that the paper can be revised.

**RESPONSE ⇒** We thank the reviewers for their helpful comments and suggestions, particularly from the perspective of researchers not already familiar with ELIXIR and its operations. We hope that the resultant additions and revisions address your concerns.

# Reviewer report: Reviewer response for version 1

**DOI:** 10.5256/f1000research.146735.r218316

**Published:** 2024-02-13

**Authors:** Patrick Comer

**Affiliations:** 1 NatureServe, Arlington, Virginia, USA

**Peer Review: The ELIXIR Biodiversity Community: Understanding short- and long-term changes in biodiversity – RM Waterhouse et al**

 This article provides a concise overview of the ELIXIR Biodiversity Community which can serve as a hub for the molecular sciences and bioinformatics as it applies to biodiversity science. ELIXIR’s overarching mission has been stated to “support the management of public research data, integrate and coordinate life science resources, and foster the development of innovative services and technical solutions in Europe” (Harrow *et al.,* 2021 ^1^). Given unprecedented and escalating loss in biodiversity, there is increasing urgency for new scientific discovery, data processing, and data sharing of biodiversity information among scientists, conservation practitioners, and policy makers. This opinion piece summarizes biodiversity threats, societal challenges and global responses, and scientific opportunities in biodiversity research. It then discusses informatics challenges facing biodiversity infrastructures and resources, organizational opportunities for ELIXIR partners, and connections with ELIXIR platforms and communities. The piece concludes with a roadmap listing key goals and long-term objectives for the ELIXIR Biodiversity Community. 

 The article might best be viewed in the context of the large and growing field of scientists, practitioners, and policy analysts, in government, academic, and non-government (NGO) sectors responding to the emerging crisis of biodiversity loss. Starting in the mid-20 ^th^ century with many academic researchers who initially documented biodiversity declines, biodiversity science - and biodiversity conservation - has been steadily mainstreamed worldwide (Locke *et al.,*2019 ^2^). One of the founders of “conservation biology” the late Michael Soulé described this science as a “discipline with a deadline” working against accelerating extinctions to sustain the wellbeing of society and the environment (Soulé and Wilcox,1980 ^3^). While encompassing the full spectrum from basic to applied, biodiversity conservation science has often emphasized practical ways to identify key information suitable for action on the ground. One good example coming from the North American NGO NatureServe network is the practical methods for systematic field inventories for natural communities and species to characterize their location, health, and status (Stein *et al.,* 2000 ^4^). In full disclosure, I have spent most of my working life in this network, first established by The Nature Conservancy in the 1970s.

 With much biodiversity conservation initially focused on identifying local areas for nature preserves, the maturing disciplines of landscape ecology, meta-population dynamics, and now climate change research, forced consideration of increasing broader scales of conservation action (Lindenmayer and Franklin, 2013 ^5^). With rapidly advancing technology and data, and emphasis on remote sensing and modeling with spatial data have become routine (Wiens *et al.,* 2009 ^6^). And increasingly (one could say belatedly), the value of social science been acknowledged as it has deepened our understanding of complex interactions of people and nature that underlay nearly all conservation strategies (Sandbrook *et al.,* 2013 ^7^).

 Therefore, while I found this piece to be factual, accurate, and well supported by the current literature, it would benefit from a broader interpretation of biodiversity science and more directed treatment of ELIXIR’s potential contributions to the “broader ecosystem” of biodiversity conservation. For example, the rapid advances in genomic science and its application to biodiversity conservation has many parallels to several allied fields previously mentioned, such as in remote sensing and several variants of the social sciences. The ELIXIR Biodiversity Community could gain by considering this prior experience. Thus, avoiding prior mistakes and replicating prior successes. Among the many ways expanding genomic science could affect biodiversity conservation include ecosystem characterization and monitoring, support to biodiversity status assessment, and evaluating progress of conservation actions. 

 The article states that “A long-term vision sees a future where sequence-based biodiversity monitoring at scale becomes the default and provides the means for ecosystem biodiversity characterisation in space and time.” One area where we may see near-term progress here is in the links between genomic data and remote sensing; where our current forms of ecosystem characterization centered on measures of species composition, ecosystem structure, and environmental setting may be enhanced by remotely-sensed data (hyperspectral  and others) indicating patterns within and among readily observed communities and species (Yamasaki *et al.,* 2017 ^8^). Relationships between measurable genetic diversity and detectable light spectra will always be complex and challenging, but with increasing frequency of remote data collection one can now envision substantial opportunities to identify leading indicators of ecosystem change through their combination.

 Current standards to assess and document endangerment status of species and ecosystems – such as those of red listing under IUCN - require documented knowledge of changing health of populations and communities (IUCN 2012 ^9^; Bland *et al.,* 2017 ^10^). These standards attempt to simplify complex ecological phenomena into standard categories to provide a reliable status assessment for use by policy makers and engaged stakeholders. Once clear health markers are identified with genomic data, this could contribute substantially to the rigor, efficiency, and impact of biodiversity status assessment.

 This naturally follows on to the potential benefit of genomic data to contribute to evaluating success (or failure) of conservation actions. As noted in the article, that these data should contribute to understanding “population-level adaptation and migration processes, in the context of anthropogenic activities ( *e.g.*, climate change and urbanisation consequences), and understand[ing] key aspects necessary to restore ecosystem functions.” It is in planning and implementation of conservation actions where one can surely recognize biodiversity conservation as inherently a human-driven process, so once again, the information used must be clear and compelling to all involved, regardless of their interests and biases.

 Prior experience in related fields suggest at least several considerations for the ELIXIR Biodiversity Community. As noted in the article, “ELIXIR can serve a critical function here, as a fundamental aspect of its mission is to make connections and coordinate across complex activities.” And “the biodiversity community needs to proactively seek common solutions (without unnecessary duplication of effort) that enable molecular technologies to advance biodiversity research. I would underscore the importance of limiting duplication of effort. Much of the history of biodiversity conservation in marked by unnecessary competition, stove-piping, and duplicated effort among those involved.

 And while ELIXIR’s “current focus is on the informatics, databases, and tools more than on the biological questions, . . . as the Community grows, it will be important to widen the diversity of its membership to ensure that the technical developments will serve the needs of biodiversity researchers,” I would encourage this community to begin early to seek out and identify feedback loops from the full spectrum of user communities. As noted above, the users of genomic data for biodiversity conservation will be diverse and demanding, so deeper consideration of user needs (Dubois *et al.,* 2020 ^11^), and participatory biodiversity science (Zoellick *et al.,* 2012 ^12^) could have substantial payoffs into the future.

 Biodiversity conservation is based on both ecological and social science that is both urgent and time sensitive. It is that applied side of the spectrum that requires urgent support from the ELIXIR Biodiversity Community.

Is the topic of the opinion article discussed accurately in the context of the current literature?

Yes

Are arguments sufficiently supported by evidence from the published literature?

Yes

Are all factual statements correct and adequately supported by citations?

Yes

Are the conclusions drawn balanced and justified on the basis of the presented arguments?

Partly

Reviewer Expertise:

Biodiversity Conservation, Landscape Ecology, Biodiversity status assessment, Climate change vulnerability and adaptation

I confirm that I have read this submission and believe that I have an appropriate level of expertise to confirm that it is of an acceptable scientific standard, however I have significant reservations, as outlined above.

## References

[1] : ELIXIR-EXCELERATE: establishing Europe's data infrastructure for the life science research of the future. *EMBO J* .2021;40(6) : 10.15252/embj.2020107409 e107409 10.15252/embj.2020107409 33565128 PMC7957415

[2] : Three global conditions for biodiversity conservation and sustainable use: an implementation framework. *Natl Sci Rev* .2019;6(6) : 10.1093/nsr/nwz136 1080-1082 10.1093/nsr/nwz136 34691979 PMC8291457

[3] : Conservation Biology: An Evolutionary-Ecological Perspective. *Sinauer* .1980;

[4] : Precious heritage: the status of biodiversity in the United States. *Oxford University Press* .2000;

[5] : Conserving forest biodiversity: a comprehensive multiscaled approach. *Island press* .2013;

[6] : Selecting and conserving lands for biodiversity: The role of remote sensing. *Remote Sensing of Environment* .2009;113(7) : 10.1016/j.rse.2008.06.020 1370-1381 10.1016/j.rse.2008.06.020

[7] : Social research and biodiversity conservation. *Conserv Biol* .2013;27(6) : 10.1111/cobi.12141 1487-90 10.1111/cobi.12141 24033825

[8] : Genomics meets remote sensing in global change studies: monitoring and predicting phenology, evolution and biodiversity. *Current Opinion in Environmental Sustainability* .2017;29: 10.1016/j.cosust.2018.03.005 177-186 10.1016/j.cosust.2018.03.005

[9] : IUCN Red List Categories and Criteria: Version 3.1. *Gland, Switzerland and Cambridge, UK* .2012;

[10] Guidelines for the application of IUCN Red List of Ecosystems Categories and Criteria. Version 1.1.2017; 10.2305/IUCN.CH.2016.RLE.3.en 10.2305/IUCN.CH.2016.RLE.3.en

[11] : Bridging the research‐implementation gap requires engagement from practitioners. *Conservation Science and Practice* .2020;2(1) : 10.1111/csp2.134 10.1111/csp2.134

[12] : "I feel like it gives me what I need to know": A qualitative study on adolescent perceptions of two contraceptive decision aids. *Contraception* .2023;128: 10.1016/j.contraception.2023.110277 110277 10.1016/j.contraception.2023.110277 37659707 PMC10999257

# Response

**Published:** 2024-05-07

**Authors:** Robert Waterhouse

**Affiliations:** University of Geneva, Switzerland

This article provides a concise overview of the ELIXIR Biodiversity Community which can serve as a hub for the molecular sciences and bioinformatics as it applies to biodiversity science. ELIXIR’s overarching mission has been stated to “support the management of public research data, integrate and coordinate life science resources, and foster the development of innovative services and technical solutions in Europe” (Harrow et al., 20211). Given unprecedented and escalating loss in biodiversity, there is increasing urgency for new scientific discovery, data processing, and data sharing of biodiversity information among scientists, conservation practitioners, and policy makers. This opinion piece summarizes biodiversity threats, societal challenges and global responses, and scientific opportunities in biodiversity research. It then discusses informatics challenges facing biodiversity infrastructures and resources, organizational opportunities for ELIXIR partners, and connections with ELIXIR platforms and communities. The piece concludes with a roadmap listing key goals and long-term objectives for the ELIXIR Biodiversity Community. 

**RESPONSE ⇒** We thank the reviewer for their positive summary.

 The article might best be viewed in the context of the large and growing field of scientists, practitioners, and policy analysts, in government, academic, and non-government (NGO) sectors responding to the emerging crisis of biodiversity loss. Starting in the mid-20th century with many academic researchers who initially documented biodiversity declines, biodiversity science - and biodiversity conservation - has been steadily mainstreamed worldwide (Locke et al.,20192). One of the founders of “conservation biology” the late Michael Soulé described this science as a “discipline with a deadline” working against accelerating extinctions to sustain the wellbeing of society and the environment (Soulé and Wilcox,19803). While encompassing the full spectrum from basic to applied, biodiversity conservation science has often emphasized practical ways to identify key information suitable for action on the ground. One good example coming from the North American NGO NatureServe network is the practical methods for systematic field inventories for natural communities and species to characterize their location, health, and status (Stein et al., 20004). In full disclosure, I have spent most of my working life in this network, first established by The Nature Conservancy in the 1970s. With much biodiversity conservation initially focused on identifying local areas for nature preserves, the maturing disciplines of landscape ecology, meta-population dynamics, and now climate change research, forced consideration of increasing broader scales of conservation action (Lindenmayer and Franklin, 20135). With rapidly advancing technology and data, and emphasis on remote sensing and modeling with spatial data have become routine (Wiens et al., 20096). And increasingly (one could say belatedly), the value of social science been acknowledged as it has deepened our understanding of complex interactions of people and nature that underlay nearly all conservation strategies (Sandbrook et al., 20137).

**RESPONSE ⇒** We thank the reviewer for the additional background, and have taken the opportunity to mention the ideas of Soulé and the field of conservation biology in the introduction.

 ADDED: “The urgency to act is recognised particularly in the field of conservation biology, which has been described as a “discipline with a deadline” (Soulé & Wilcox 1980).”

 Therefore, while I found this piece to be factual, accurate, and well supported by the current literature, it would benefit from a broader interpretation of biodiversity science and more directed treatment of ELIXIR’s potential contributions to the “broader ecosystem” of biodiversity conservation. For example, the rapid advances in genomic science and its application to biodiversity conservation has many parallels to several allied fields previously mentioned, such as in remote sensing and several variants of the social sciences. The ELIXIR Biodiversity Community could gain by considering this prior experience. Thus, avoiding prior mistakes and replicating prior successes. Among the many ways expanding genomic science could affect biodiversity conservation include ecosystem characterization and monitoring, support to biodiversity status assessment, and evaluating progress of conservation actions. 

**RESPONSE ⇒ **While, we agree with the reviewer’s ideas to highlight better the “broader ecosystem” of biodiversity conservation,the role of the ELIXIR Biodiversity Community has been clearly defined in the section “Organisational opportunities and ELIXIR’s roles” as we recognise that there is a plethora of initiatives already tackling exactly these kinds of issues. Rather, the Community is  focused on the data management aspects, in particular with regards to molecular data, that are needed to effectively support and grow the many existing activities in the domain of biomonitoring and ecosystem health. We have made changes to the text to ensure that there is clarity to the role that the ELIXIR Biodiversity Community plays (data science support versus frontline conservation actions). 

 The article states that “A long-term vision sees a future where sequence-based biodiversity monitoring at scale becomes the default and provides the means for ecosystem biodiversity characterisation in space and time.” One area where we may see near-term progress here is in the links between genomic data and remote sensing; where our current forms of ecosystem characterization centered on measures of species composition, ecosystem structure, and environmental setting may be enhanced by remotely-sensed data (hyperspectral  and others) indicating patterns within and among readily observed communities and species (Yamasaki et al., 20178). Relationships between measurable genetic diversity and detectable light spectra will always be complex and challenging, but with increasing frequency of remote data collection one can now envision substantial opportunities to identify leading indicators of ecosystem change through their combination.

**RESPONSE ⇒** This example is an excellent case study of how different data types - if made available and interoperable - can be co-interrogated to improve how we measure and monitor biodiversity. We have revised the text to more clearly indicate the complementarity of different types of data and their applications.

 EDITED ⇒ A long-term vision sees a future where sequence-based biodiversity monitoring at scale becomes a default and provides the means for ecosystem biodiversity characterisation in space and time, complemented and enhanced by other biomonitoring technologies. 

 Current standards to assess and document endangerment status of species and ecosystems – such as those of red listing under IUCN - require documented knowledge of changing health of populations and communities (IUCN 20129; Bland et al., 201710). These standards attempt to simplify complex ecological phenomena into standard categories to provide a reliable status assessment for use by policy makers and engaged stakeholders. Once clear health markers are identified with genomic data, this could contribute substantially to the rigor, efficiency, and impact of biodiversity status assessment. This naturally follows on to the potential benefit of genomic data to contribute to evaluating success (or failure) of conservation actions. As noted in the article, that these data should contribute to understanding “population-level adaptation and migration processes, in the context of anthropogenic activities (e.g., climate change and urbanisation consequences), and understand[ing] key aspects necessary to restore ecosystem functions.” It is in planning and implementation of conservation actions where one can surely recognize biodiversity conservation as inherently a human-driven process, so once again, the information used must be clear and compelling to all involved, regardless of their interests and biases.

**RESPONSE ⇒ **We agree that this is a key goal in biodiversity genomics, being able to get the most out of the data to improve the way assessments are made and how to interpret the results for policy makers and engaged stakeholders. We see the role of ELIXIR here as focused on helping to build systems that will ensure access to data and tools and services that enable experts to perform assessments and make decisions based on the best available data and methods. We have made changes to ensure clarity to this distinction (data science support versus frontline conservation actions).

 Prior experience in related fields suggest at least several considerations for the ELIXIR Biodiversity Community. As noted in the article, “ELIXIR can serve a critical function here, as a fundamental aspect of its mission is to make connections and coordinate across complex activities.” And “the biodiversity community needs to proactively seek common solutions (without unnecessary duplication of effort) that enable molecular technologies to advance biodiversity research. I would underscore the importance of limiting duplication of effort. Much of the history of biodiversity conservation in marked by unnecessary competition, stove-piping, and duplicated effort among those involved.

**RESPONSE ⇒** We thank the reviewer for highlighting this important point. One of the strengths of ELIXIR’s operations and activities in other domains like human health has been to contribute to better coordinated efforts across Europe. It is a cornerstone of the principle of distributed infrastructure for life-science data and we have incorporated your suggestions. 

 ADDED ⇒ A key part of this is the building of distributed infrastructures for life-science data that avoid or minimise unnecessary duplication of effort to be able to advance efficiently towards common goals. 

 And while ELIXIR’s “current focus is on the informatics, databases, and tools more than on the biological questions, . . . as the Community grows, it will be important to widen the diversity of its membership to ensure that the technical developments will serve the needs of biodiversity researchers,” I would encourage this community to begin early to seek out and identify feedback loops from the full spectrum of user communities. As noted above, the users of genomic data for biodiversity conservation will be diverse and demanding, so deeper consideration of user needs (Dubois et al., 202011), and participatory biodiversity science (Zoellick et al., 201212) could have substantial payoffs into the future. Biodiversity conservation is based on both ecological and social science that is both urgent and time sensitive. It is that applied side of the spectrum that requires urgent support from the ELIXIR Biodiversity Community.

**RESPONSE ⇒** Since this manuscript was first published the Community has embarked on its first Implementation Study, within which there is a dedicated effort to describe the landscape of stakeholders ELIXIR is working with or needs to better engage with and to establish a functional “network of networks” for biodiversity research. Following your suggestions, we have revised the last section of the manuscript, highlighting the needs to engage beyond ELIXIR members and interact with other communities of practice in the domain.

 ADDED ⇒ Beyond the ELIXIR Biodiversity Community itself, it is also vital to engage with other communities in the domain, including with stakeholders such as practitioners and citizen science initiatives in order to contribute towards bridging the gaps between research and implementation (Dubois et al., 2020; Fraisl et al. 2022).
